# Antisense Oligonucleotide Activation via Enzymatic Antibiotic Resistance Mechanism

**DOI:** 10.1021/acschembio.3c00027

**Published:** 2023-06-16

**Authors:** Kristie E. Darrah, Savannah Albright, Rohan Kumbhare, Michael Tsang, James K. Chen, Alexander Deiters

**Affiliations:** † Department of Chemistry, University of Pittsburgh, Pittsburgh, Pennsylvania 15260, United States; ‡ Department of Developmental Biology, University of Pittsburgh, Pittsburgh, Pennsylvania 15260, United States; § Department of Chemical and Systems Biology, Stanford University School of Medicine, Stanford, California 94305, United States

## Abstract

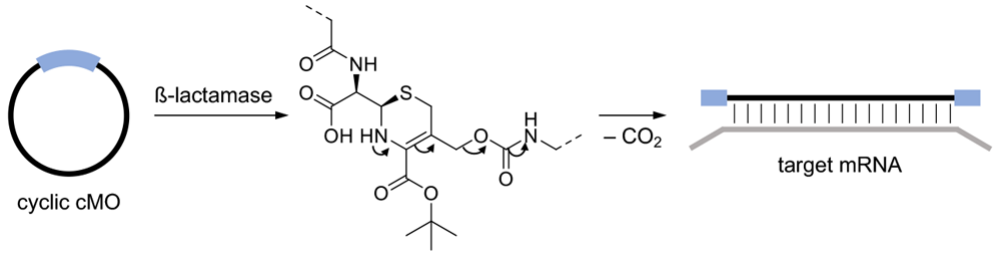

The structure and mechanism of the bacterial enzyme β-lactamase have been well-studied due to its clinical role in antibiotic resistance. β-Lactamase is known to hydrolyze the β-lactam ring of the cephalosporin scaffold, allowing a spontaneous self-immolation to occur. Previously, cephalosporin-based sensors have been developed to evaluate β-lactamase expression in both mammalian cells and zebrafish embryos. Here, we present a circular caged morpholino oligonucleotide (cMO) activated by β-lactamase-mediated cleavage of a cephalosporin motif capable of silencing the expression of T-box transcription factor Ta (*tbxta*), also referred to as *no tail a* (*ntla*), eliciting a distinct, observable phenotype. We explore the use of β-lactamase to elicit a biological response in aquatic embryos for the first time and expand the utility of cephalosporin as a cleavable linker beyond targeting antibiotic-resistant bacteria. The addition of β-lactamase to the current suite of enzymatic triggers presents unique opportunities for robust, orthogonal control over endogenous gene expression in a spatially resolved manner.

## Introduction

Nucleic acid-based tools have found widespread application in the study of gene function, e.g., as antisense agents, due to their ease of synthesis, programmability, and specificity.^1−6^ A drawback of oligonucleotides can be their susceptibility to nuclease-mediated degradation, which can be addressed through the incorporation of chemical modifications.^1^ Morpholino oligonucleotides (MOs) are one class of modified nucleic acid analogs, containing a six-membered morpholine ring in place of the ribose and a neutral phosphoramidite linkage within the backbone. Despite the structural differences from natural nucleic acids, MOs maintain their high specificity and affinity to hybridize to the corresponding target RNA sequences.^7,8^ MOs allow for gene silencing by blocking translation or mRNA splicing.^9−12^

MOs are frequently used to probe both maternal and zygotic gene function during embryonic development in zebrafish embryos, as delivery into a fertilized oocyte results in immediate and global gene silencing with effects persisting up to 5 days post-fertilization.^13−15^ The development of caged MOs (cMOs) that can be activated with external triggers has broadened the utility of these tools for analysis of dynamic cellular processes with precise spatial and temporal control. This is particularly important for investigations of gene function during later developmental stages or tissue-specific cellular processes, both of which are impeded by immediate, global silencing. Upon delivery, cMOs are functionally inert and unable to engage their target sequences until activated with an external trigger, imparting conditional control over MO-induced gene silencing.^16^ We and others have devised various MO caging strategies by incorporating light-,^17−22^ enzyme-,^23^ and more recently, small molecule-responsive elements^24^ within the MO structure. Multiple designs have resulted in successful caging and subsequent conditional control of silencing function, including the incorporation of cleavable groups onto nucleobases,^18,19^ short, complementary blocking oligonucleotides that form hairpins or duplexes,^17,25−27^ and linkers for macrocyclization.^20−24,28−33^ While all of these designs have demonstrated varying degrees of success in aquatic embryo systems, end-to-end macrocyclization has proven to be a highly modular approach that can be used with a wide range of immolative linkers^34^ and commercially available MOs bearing suitable reactive handles. The curvature induced by the cyclization (and more recently bicyclization)^20^ of the MO impedes target binding until a single cleavage event restores the active, linear species, which can then hybridize to its mRNA target and induce gene silencing.

Enzymatic triggers provide unique opportunities for conditional control that complement current light- and small molecule-activated cMOs. As an example, precise activation by light exposure can be complicated by the inherent structural complexity, opacity, and physical movement of individual cells within developing organisms. In the case of small molecule delivery, controlled activation of the MO can be limited by diffusion of the small molecule trigger. These shortcomings can be addressed through enzymatic activation, as the triggering enzyme can be expressed in specific cells and at controlled time points through the use of inducible or tissue-specific promoters.^35,36^ The Chen lab has demonstrated this previously using an *Escherichia coli* nitroreductase, *Nfsb*, to control the activity of a cyclic cMO tethered with a 4-nitrobenzyl linker in live zebrafish embryos.^23^ Additionally, the Dmochowski group has developed a caspase-3 activatable peptide nucleic acid (PNA)-based oligonucleotide sensor to probe apoptosis in cells.^37^ Taken together, these reports demonstrate the versatility of enzyme-mediated strategies that can be applied to nucleic acid-based tools.

Herein, we expand upon this previous work and demonstrate for the first time the use of the bacterial enzyme β-lactamase to control oligonucleotide function. The use of β-lactamase as a triggering enzyme is particularly appealing since the structure, mechanism, and substrate specificity are well-established due to the enzyme’s prominent role in antibiotic resistance.^38,39^ β-Lactamases also possess many other favorable characteristics, including stability, catalytic efficiency, and the fact that their enzymatic function is unique to bacterial systems. As a result of this biorthogonality to the mammalian cellular environment, β-lactamases have been used to spatially control the activity of various drugs in directed enzyme prodrug therapy (DEPT) strategies.^38−42^ Cephalosporin motifs have been previously functionalized for β-lactamase-mediated activation of prodrugs against antibiotic resistant bacterial strains^43,44^ and fluorescent sensors probing β-lactamase expression in mammalian cells and zebrafish embryos.^45,46^ Here, we leverage β-lactamase-mediated antibiotic resistance mechanisms to control MO silencing function *in vivo* by designing and synthesizing a cyclic cMO tethered by a cephalosporin-based linker that is efficiently cleaved via β-lactamase-mediated hydrolysis.

## Results and Discussion

**Figure 1:**
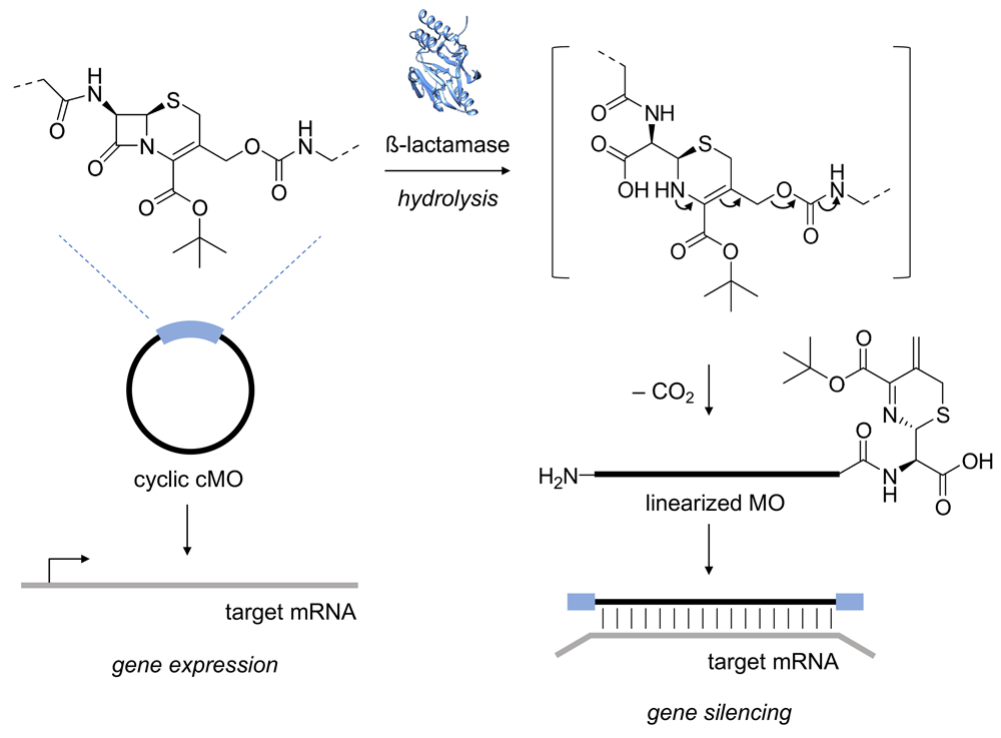
Following enzyme-mediated hydrolysis of the β-lactam ring by β-lactamase (PDB ID 4F6H), the resulting amine intermediate undergoes self-immolation. The release of the 5′-end generates the active, linearized MO capable of hybridizing to its respective target mRNA and silencing gene expression.

Upon β-lactamase catalysis, the core β-lactam ring of the linker is hydrolyzed, generating the corresponding amine, which then undergoes self-immolation and spontaneous elimination via the carbamate linkage. This elimination reaction releases the 5′-terminus of the MO to generate the active, linear MO capable of hybridizing to the mRNA target and subsequently inducing gene silencing (Figure 1).

To synthesize the β-lactamase-activated cMO, the linker **8** was designed using a cephalosporin core scaffold. The C-3 and C-7 positions of the core cephalosporin scaffold were synthetically modified with orthogonal azido and chloroacetamide handles, respectively, necessary for bioconjugation to the terminally modified linear MO.^39^ The carbamate linkage was strategically installed at the C-3 position as it has been previously shown that leaving groups attached to the exocyclic methylene can undergo spontaneous elimination following hydrolysis of the β-lactam ring.^47,48^

**Figure 2:**
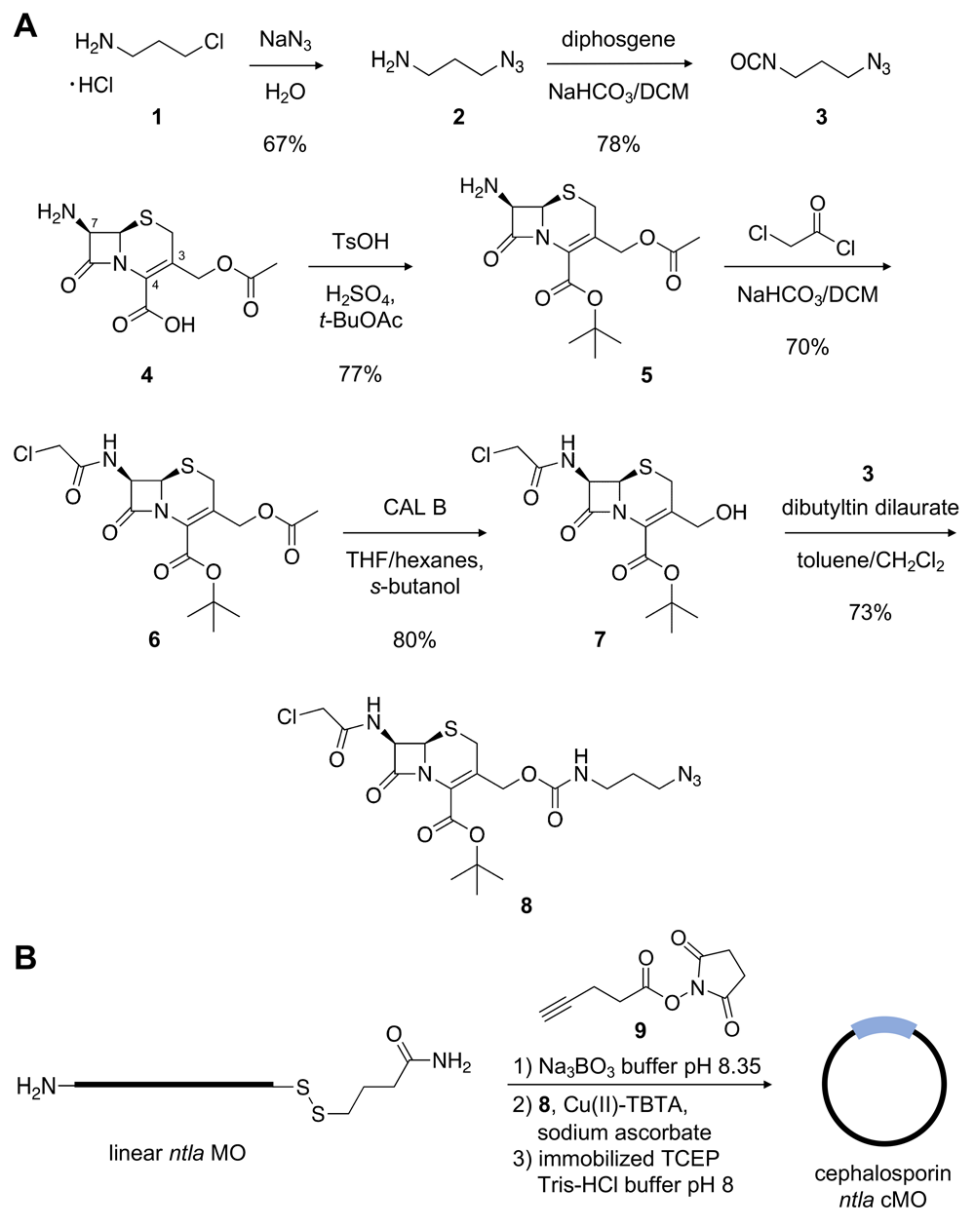
(A) Synthetic scheme of the cephalosporin-containing linker **8**. (B) Synthesis of the cephalosporin *ntla* cMO.

The linker **8** was synthesized in four steps from commercially available 7-aminocephalosporanic acid (**4**) (Figure 2A). The *tert*-butyl ester **5** was obtained by treating **4** with *tert*-butyl acetate in the presence of *p*-toluenesulfonic acid (TsOH) and concentrated sulfuric acid.^49^ The chloroacetamide at the C-7 position was installed by reacting the free amine **5** with 2-chloroacetyl chloride to form the corresponding amide **6**. Selective cleavage of the acetate at the C-3 position was achieved chemoenzymatically through lipase-mediated hydrolysis of **6** using the serine hydrolase *Candida antarctica* lipase B (CAL B) to generate the free alcohol **7**.^49^ To install the azide handle, 3-azidopropan-1-amine (**2**) was synthesized from the corresponding alkyl chloride **1** as previously reported.^50^ The amine **2** was converted to the corresponding isocyanate **3** through treatment with diphosgene. The isocyanate **3** was then added to a solution of the alcohol **7** in the presence of dibutyltin dilaurate to generate the corresponding carbamate-containing cephalosporin linker **8**. It should be noted that while the canonical cephalosporin scaffold contains a carboxylic acid moiety at the C-4 position, there have been no direct studies of enzyme reactivity with any synthetic C-4-protected analogs. To test that the bulky *tert*-butyl ester in **8** still presents a suitable enzyme substrate, the linker (1 mM) was incubated with recombinant β-lactamase (0.5 μg), and the reaction progression was monitored by LCMS (Supporting Figure S1). Gratifyingly, nearly full cleavage of the linker **8** was observed after 3 h, and the expected cleavage product was generated. These results suggest that the presence of the *tert*-butyl ester at the C-4 position does not impede enzymatic cleavage of the lactam ring. With knowledge that the linker can be cleaved by β-lactamase, we moved forward with the macrocyclization of our desired MO with the functionalized linker **8**.

A cyclic cephalosporin cMO targeting the T-box transcription factor Ta (*tbxta*), also known as *no tail a* (*ntla*, 5′-GACTTGAGGCAGCATATTTCCGAT-3′, anti-start codon underlined) was prepared in three steps using the synthesized linker **8** (Figure 2B). MO-mediated silencing of *ntla* during early embryogenesis induces an obvious and distinct morphant phenotype that includes truncation of the embryo tail and misshapen somites.^51,52^ Further, we and others have previously utilized the *ntla* MO as a model gene target for validating other conditionally activated cMO technologies.^17,22−24,27−29,31^ The *ntla* MO, containing 5′-amine and 3′-disulfide handles, was subjected to macrocyclization^28,29^ by first acylating the terminal amine with the NHS ester **9** to yield the corresponding alkyne suitable for bioconjugation to the azide-containing linker. The linker **8** was clicked to the alkyne-functionalized *ntla* MO via a copper(I)-catalyzed alkyne–azide cycloaddition (CuAAC).^53^ Lastly, the 3′-disulfide was reduced in the presence of resin-immobilized tris(2-carboxyethyl)phosphine (TCEP) to generate the free thiol, which then undergoes a spontaneous intramolecular cyclization via thioether formation with the chloroacetamide handle of **8** to generate the cyclic cephalosporin *ntla* cMO. Reaction progress for each step was analyzed via MALDI-TOF MS and purified by high-performance liquid chromatography (HPLC) (Supporting Figure S2). To ensure the removal of any remaining linear MO species, the final cMO product was subject to additional purification steps using iodoacetyl- and NHS-functionalized resins, as previously described.^24^

**Figure 3:**
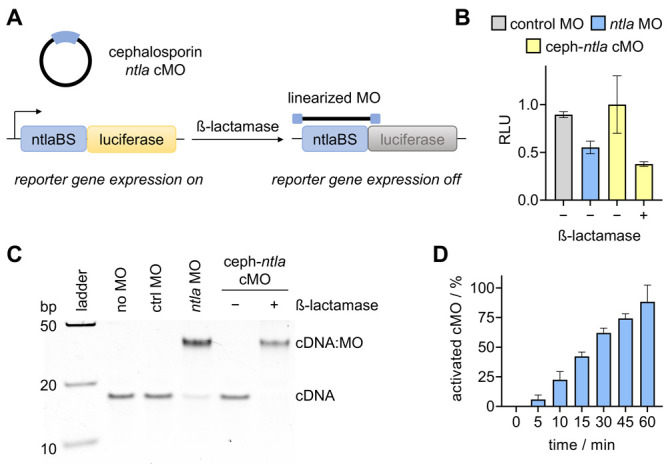
(A) Luciferase reporter assay in which luciferase expression is silenced in the presence of linearized MO. (B) Luminescence signal after treatment of the indicated MO. Data represent average ± SEM from three independent experiments. (C) Gel-shift assay showing activation of the circular morpholino sequestering its complementary target DNA (cDNA) in the presence of β-lactamase. (D) Quantification of a time course analysis of cyclic cephalosporin *ntla* cMO cleavage following incubation with recombinant β-lactamase. Bars represent averages and error bars represent standard deviations from three independent experiments.

After confirmation of successful macrocyclization, we next sought to evaluate enzyme-mediated linearization and activation of the cephalosporin *ntla* cMO. Linearization was performed by incubating the cMO (20 μΜ) with recombinant β-lactamase for 3 h. We utilized a reticulocyte lysate-based translation system to analyze activation of the cMO.^24^ The consensus *ntla* binding sequence (ntlaBS) was cloned directly upstream of and in frame with the coding sequence of the firefly luciferase reporter gene. Hybridization of the MO to this target sequence blocks the translational machinery from accessing the start site, thereby repressing luciferase expression (Figure 3A). Following treatment with linear *ntla* MO (1 μM), luciferase expression is reduced by 50%, whereas treatment with the cyclic cephalosporin *ntla* cMO did not impact luciferase levels. However, MO silencing function is fully restored following linearization of the cyclic cMO with β-lactamase, as evidenced by a nearly 65% reduction of reporter gene expression (Figure 3B). Taken together, this data suggests that enzymatic catalysis by β-lactamase can be used as an efficient off to on switch to conditionally control MO gene silencing activity.

While switching of MO activity looked excellent, we were surprised by the overall limited silencing of luciferase expression in the *in vitro* translation assay and thus conducted a gel-shift analysis of the MO:target interaction. A 25-mer DNA oligonucleotide that is fully complementary to the *ntla* MO (cDNA, Supporting Table S2) was used as an mRNA mimic. Heteroduplex formation with the MO or cMO was visualized by native PAGE (Figure 3C). No heteroduplex formation was observed with the cMO in the absence of β-lactamase, confirming the results of the *in vitro* translation assay, as the curvature induced by macrocyclization of the cMO prevents target binding. It is only after incubation with the triggering enzyme that nearly full sequestration of the target cDNA is observed, as evidenced by the shift in mobility of the heteroduplex in the gel. This shift indicates successful cleavage of the linker and subsequent target hybridization by the decaged linear MO. To quantitatively determine the activated amount of cMO, the circular antisense agent was incubated with recombinant β-lactamase for up to 1 h. At defined time points, the enzyme was heat-inactivated and the MO was hybridized with an excess of cDNA. The band densities in a gel-shift assay were quantified using a standard curve (Supporting Figure S3) to determine the percentage of activated cMO. After 1 h, the cMO was 88% linearized (Figure 3D and Supporting Figure S4). The nearly complete heteroduplex formation observed by the decaged MO suggested promise for *in vivo* gene silencing.

**Figure 4:**
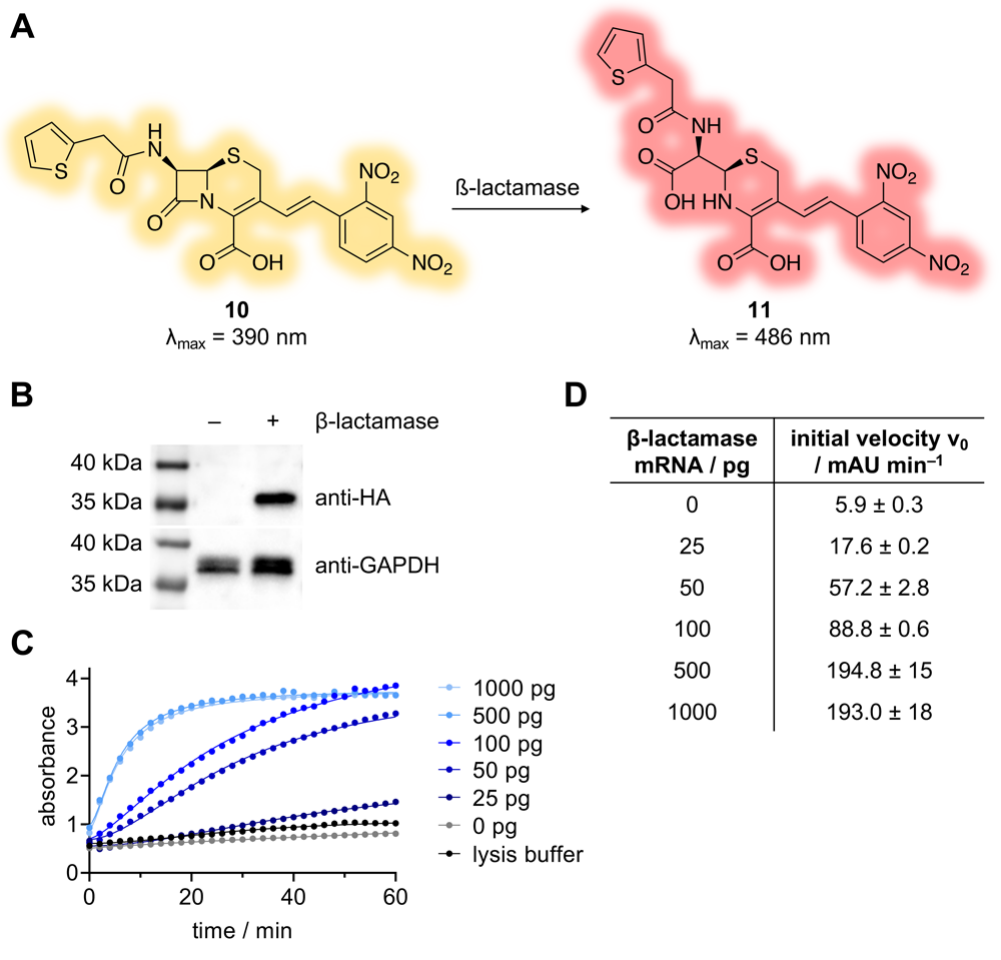
(A) Structure of the chromogenic β-lactamase substrate nitrocefin (**10**), which is hydrolyzed to **11** following β-lactamase catalysis. (B) Western blot analysis of β-lactamase expression in embryos injected with 400 pg of mRNA. (C) Zebrafish lysates analyzed for β-lactamase activity by monitoring production of **11** (absorbance at 486 nm) over time. (D) Initial velocities were determined through a linear regression analysis of the first 10 min of the data presented in (C). The error values indicate the standard deviation of values for the best fit parameters from the linear regression analysis.

Next, we sought to investigate whether β-lactamase could be used to control MO-mediated gene silencing of an endogenous gene target during early zebrafish development. β-lactamase activity can be easily evaluated using nitrocefin (**10**), a cephalosporin-based chromogenic substrate that changes in color from yellow to red following β-lactam hydrolysis (yielding **11**; Figure 4A).^54^ The utility of this substrate was validated using recombinant β-lactamase (Supporting Figure S5). We generated a β-lactamase expression construct by cloning an N-terminally HA-tagged mammalian codon-optimized β-lactamase into the pCS2+ vector, which can be used for enzyme expression in mammalian cells and as a template for *in vitro* mRNA production (Supporting Table S2).^55^ The expression of β-lactamase was confirmed in HEK293T cells and enzymatic function was validated by monitoring cleavage of the nitrocefin substrate (Supporting Figure S6).

In order to evaluate β-lactamase expression and activity *in vivo*, we next generated synthetic β-lactamase mRNA via *in vitro* transcription (Supporting Figure S7). The synthetic β-lactamase mRNA was injected into the yolk sac of 1- to 4-cell-stage zebrafish embryos. At 24 h post-fertilization (hpf), the injected embryos were collected, lysed,^56^ and β-lactamase expression was confirmed by Western blot (Figure 4B). To confirm that β-lactamase also maintained hydrolase activity, embryos were injected with increasing amounts of mRNA, lysed, and then incubated with nitrocefin. Hydrolysis of the substrate was monitored over time. An obvious dose-dependent correlation in β-lactamase activity was observed (Figure 4C). Further, quantification of the initial rates of nitrocefin hydrolysis demonstrated a clear positive correlation between the amount of mRNA injected into the embryos and the rate of enzymatic activity (Figure 4D). These results suggest that the extent of enzymatic activity can be finely tuned with the mRNA injection amount, with maximal activity plateauing following injection of approximately 500 pg of β-lactamase mRNA.

**Figure 5:**
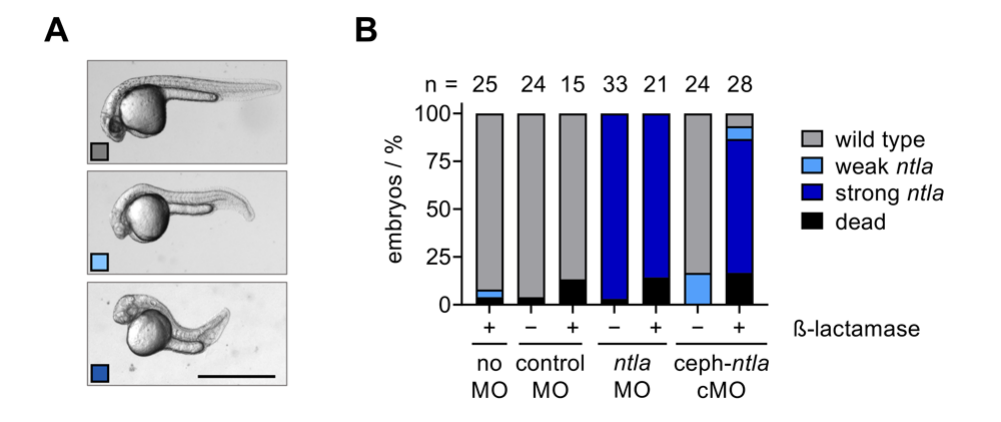
(A) Representative images of *ntla* morphant phenotypes in zebrafish embryos at 24 hpf. Scale bar equals 1 mm. (B) Phenotypic scoring of embryos injected with the indicated morpholino and amount of β-lactamase mRNA.

We then utilized our cyclic cephalosporin *ntla* cMO to determine whether we could induce enzymatic activation of MO function *in vivo*. As mentioned previously, *ntla* has been established as an excellent candidate gene target for silencing using conditionally controlled MO technologies.^17,23,27−29^ Silencing of *ntla* expression during early embryogenesis results in a distinct phenotype observable at 24 hpf, including truncation of tail length, loss of the notochord, and U-shaped somites (Figure 5A).^51,52^ The quantification of embryo body length was used to evaluate the strength of the morphant phenotype (Supporting Figure S8).^24^ Zebrafish embryos were injected in the yolk with 200 pg of either a negative control MO, the linear *ntla* MO, or our cyclic cephalosporin *ntla* cMO following previously established methods for zebrafish microinjections.^14,56,57^ Based on the diameter (0.7 mm) and cytoplasmic bridging of early-stage embryos, we estimate the final concentration of the morpholino to be approximately 120 nM in the injected embryos.^58,59^ Microinjection into the embryo yolk sac, as opposed to directly into the cell, enables a higher throughput workflow (200-300 embryos from one mating)^60^ and reduces injection errors that could result in embryo-to-embryo variation. However, a caveat of this injection technique is that precise assessment of the intracellular MO concentration is difficult to discern.^59^ Where indicated, 400 pg of β-lactamase mRNA was coinjected with the specified MO or cMO, and embryos were incubated until 24 hpf (Figure 5B). Embryos that were not injected or injected with the control MO developed as expected and displayed minimal signs of toxicity. However, we observed a slight increase in toxicity in embryos coinjected with MO and mRNA. To further investigate this toxicity, control MO (200 pg) was injected with increasing amounts of β-lactamase mRNA (up to 1000 pg). No trend was detected and no increase in phenotypic defects or toxicity was observed (Supporting Figure S9). Importantly, embryos injected with the cyclic cephalosporin *ntla* cMO alone also exhibited minimal phenotypic defects, confirming the inactivity of the cyclic cMO *in vivo*. However, upon coinjection of the cephalosporin *ntla* cMO with β-lactamase mRNA, full rescue of the *ntla* morphant phenotype was observed, comparable to the frequency of phenotype observed with the linear *ntla* MO. Gratifyingly, zebrafish coinjected with the cyclic cephalosporin *ntla* cMO and synthetic *Nfsb* mRNA did not show any *ntla* morphant phenotypes (Supporting Figure S10A). The same observation was made when injected embryos were exposed to 365 or 405 nm light (Supporting Figure S10B). Together, these results demonstrate the orthogonality of the two enzymatic cMO activation approaches and to the optical stimulation used for photocaged cMOs. There was a higher frequency of nonspecific phenotypic defects in *Nfsb*-mRNA injected embryos, showing lower toxicity of the β-lactamase expression (Supporting Figures 9A and 10A).^52^ Taken together, these results support the utility of the β-lactamase enzyme for conditionally controlling cephalosporin-modified antisense oligonucleotides to investigate endogenous gene function *in vivo*.

## Conclusion

In summary, we leveraged enzyme-mediated antibiotic resistance mechanisms to develop a β-lactamase-triggered antisense agent. A novel linker containing a cephalosporin core-scaffold was synthesized and used to cyclize a MO. The curvature of the cyclic cMO successfully abrogated hybridization to and silencing of its mRNA target. β-lactamase-mediated cleavage of the cMO linker, MO linearization, and restoration of gene silencing were confirmed *in vitro* by gel-shift assay and silencing of reporter gene expression. Efficient enzyme-triggered silencing of endogenous gene expression was demonstrated in live zebrafish embryos coinjected with the cyclic cephalosporin *ntla* cMO and mRNA expressing β-lactamase. These results complement the current suite of conditionally controlled nucleic acids.^17−20,22−24,27−29,31^ Despite only one prior example of β-lactamase use in zebrafish embryos,^45^ the work presented herein demonstrates that the highly specific interaction between β-lactamase and cephalosporin provides a novel and robust means to control gene expression in a potentially tunable manner. Future studies placing β-lactamase expression under the control of tissue-specific promoters (similar to what has been shown previously with nitroreductase to enable targeted cell ablation experiments)^23^ will provide spatial and temporal control of enzyme function. While numerous fish lines expressing nitroreductase have been reported,^61^ transgenic fish lines stably expressing β-lactamase have not yet been generated, thereby precluding these studies. We anticipate that the enzyme activation presented here could be multiplexable with the other available orthogonal approaches for the conditional control of MO function.^19,20,22−24,28,29^ Lastly, the activation of MOs demonstrates that β-lactamase expression has potential for being a broadly applicable trigger for molecular function in zebrafish, significantly expanding its utility.

## Supporting Information Available

Detailed experimental protocols and supporting data and figures (PDF)
