# Supplementary material for: Antisense Oligonucleotide Activation via Enzymatic Antibiotic Resistance Mechanism
Source: ACS Chem Biol. 2023 Jun 16;18(10):2176–82. doi: 10.1021/acschembio.3c00027 (PMC10592181; doi:10.1021/acschembio.3c00027)
Supplement: Supplementary file 1 — cb3c00027_si_001.pdf [file cb3c00027_si_001.pdf]

Supporting Information

Antisense Oligonucleotide Activation via Enzymatic Antibiotic Resistance Mechanism

Kristie Darrah[a], Savannah Albright[a], Rohan Kumbhare[a], Michael Tsang[b], James K. Chen[c], and Alexander Deiters[a]

[a]Department of Chemistry, University of Pittsburgh, Pittsburgh, PA 15260, United States

[b]Department of Developmental Biology, School of Medicine, University of Pittsburgh, Pittsburgh, PA 15260, United States

[c]Department of Chemical and Systems Biology, Stanford University School of Medicine, Stanford, CA 94305, United States

Supporting Figures and Tables

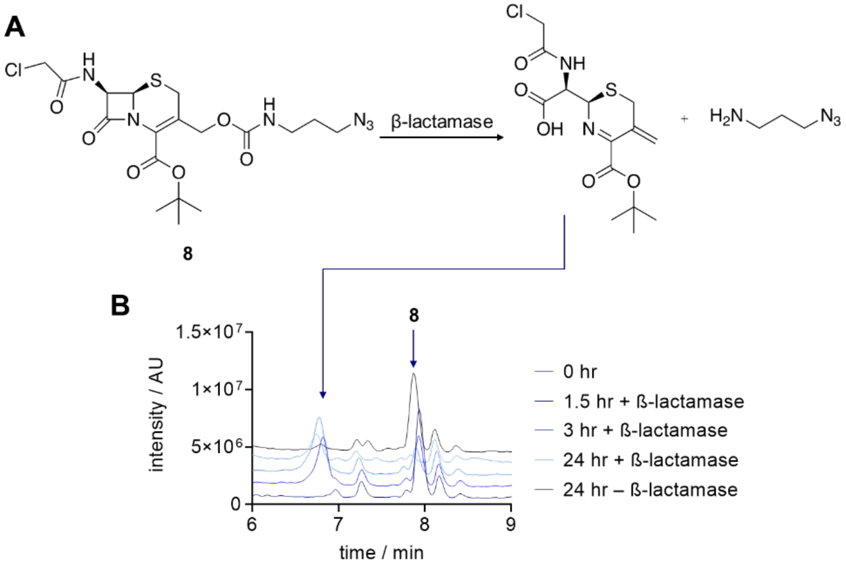

Supporting Figure S1. Cleavage and analysis of *t*Bu-cephalosporin linker 8. A) Reaction scheme of *t*Bu-cephalosporin *ntla* cMO linker **8** to form cleavage products **S1** and **S2** following hydrolysis by ß-lactamase. B) Time course analysis of the cleavage of **8** by recombinant ß-lactamase enzyme as monitored by LCMS. Linker **8** (1 mM) was incubated with recombinant ß-lactamase (1 µg) and reaction progression was monitored over 24 hours by LCMS (ESI).

A

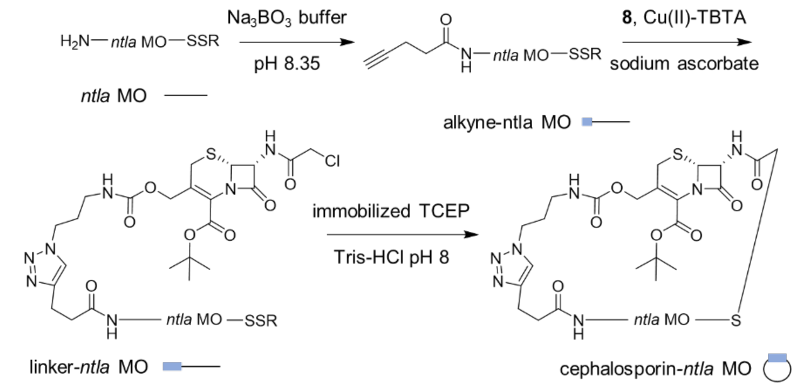

B

	calculated	observed
ntla MO	8968.89 Da	8967.78 Da
alkyne *ntla* MO	9048.97 Da	9048.03 Da
linker *ntla* MO	9537.92 Da	9539.16 Da
cephalosporin *ntla* cMO	9384.29 Da	9381.82 Da

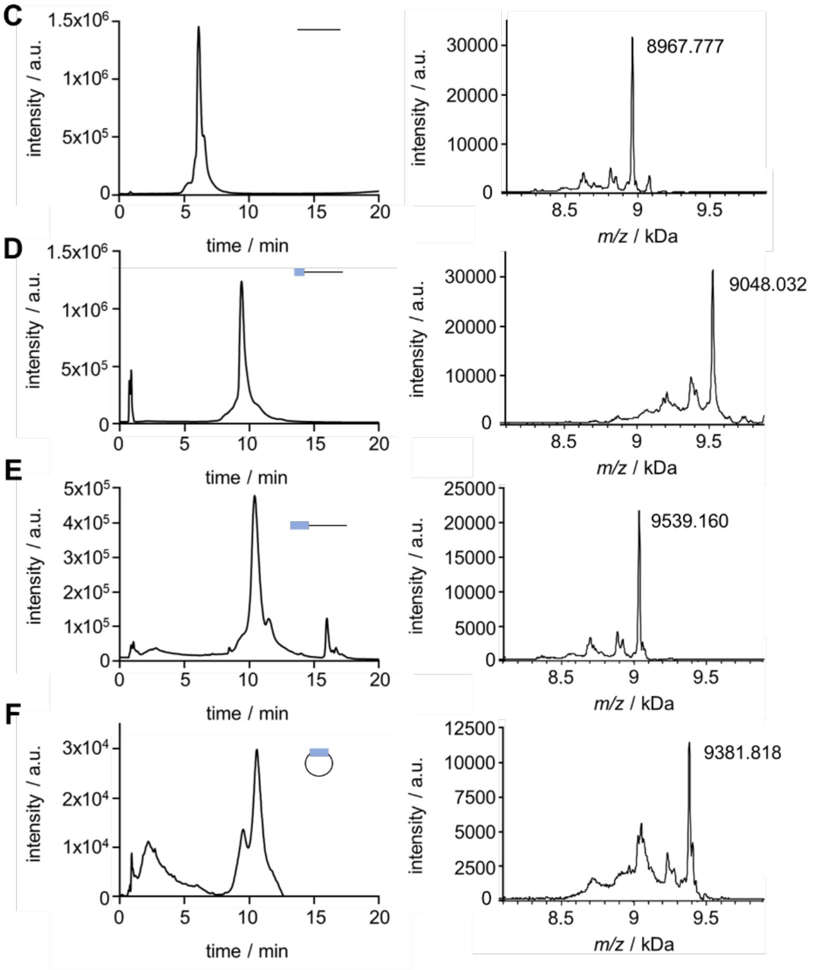

Supporting Figure S2. Synthesis of the cephalosporin *ntla* cMO. A) Reaction scheme of *ntla* MO macrocyclization with the cephalosporin linker. B) MALDI-TOF MS masses of key reaction species presented in panel **A**. Representative HPLC chromatograms and MALDI-TOF MS spectra of C) *ntla* MO, D) alkyne-*ntla* MO, E) cephalosporin linker *ntla* MO, and F) cephalosporin *ntla* cMO.

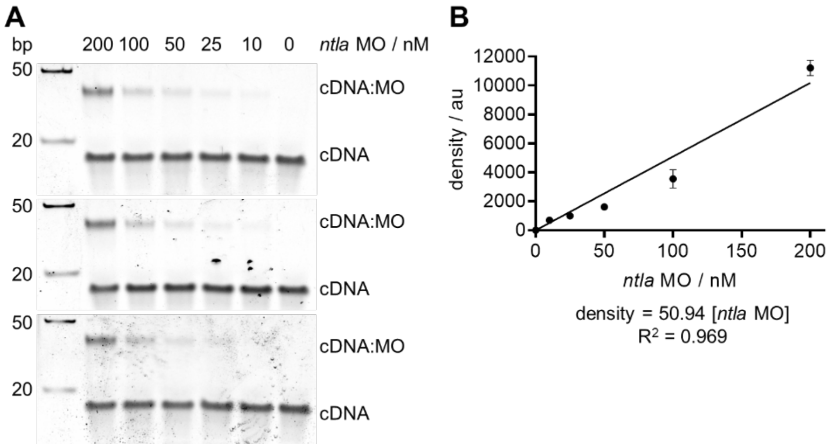

Supporting Figure S3. Generation of a standard curve for heteroduplex formation. A) Gel-shift assays showing cDNA:MO duplex formation. An excess of complementary *ntla* DNA (cDNA) was incubated with the indicated concentrations of linear *ntla* MO. B) Standard curve representing quantification of the integrated raw density values of the cDNA:MO duplex. Integration was performed in ImageJ. Data points represent the average raw density and error bars represent standard deviation from three independent experiments.

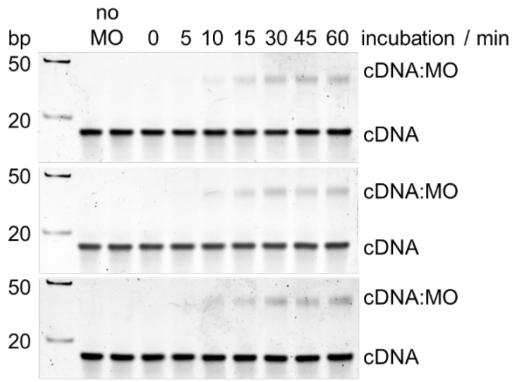

Supporting Figure S4. Time course analysis of cephalosporin *ntla* cMO cleavage by gel-shift assay. The cephalosporin *ntla* cMO was incubated with recombinant ß-lactamase for the indicated amount of time before heat inactivation. The cMO was then incubated with an excess of complementary *ntla* cDNA and analyzed for duplex formation via gel-shift assays. Extent of cleavage was determined by quantifying the integrated raw density values and extrapolating from the standard curve presented in Supporting Figure S3.

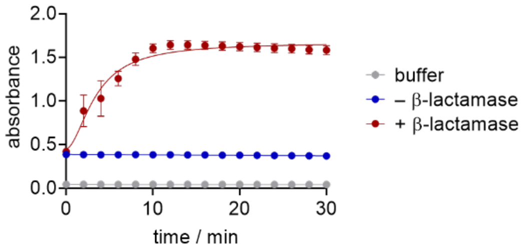

Supporting Figure S5. Nitrocefin-based assay of ß-lactamase activity. Cleavage of nitrocefin was monitored by measuring absorbance at 486 nm over time. Full cleavage of nitrocefin was observed when incubated with recombinant ß-lactamase.

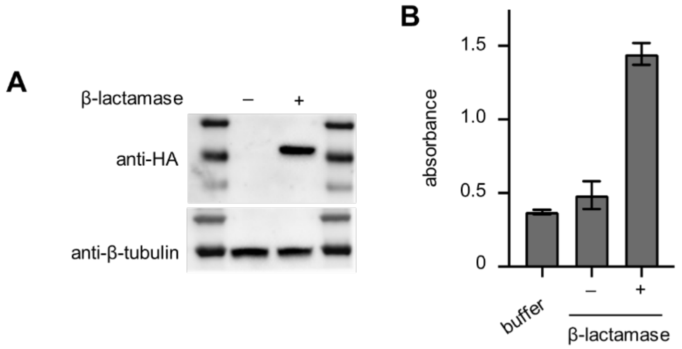

Supporting Figure S6. Evaluation of ß-Lactamase expression and activity *in vitro*. A) Western blot analysis of HEK293T lysates that were transiently transfected with pCS2-HA-ß-lactamase. L = ladder. B) Nitrocefin hydrolysis by HEK293T cell lysates transfected with pCS2-HA-ß-lactamase and incubated with **10** for 2 minutes (measured absorbance at 486 nm). Data represents averages ± standard deviation from three independent experiments.

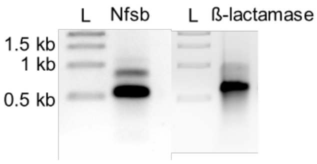

Supporting Figure S7. Gel analysis of *in vitro* transcribed mRNA. *Nfsb* and ß-lactamase mRNA products were analyzed on a 0.8% agarose gel following synthesis by *in vitro* transcription. mRNA products may resolve as two bands due to secondary structure of the synthesized transcript. L = ladder.

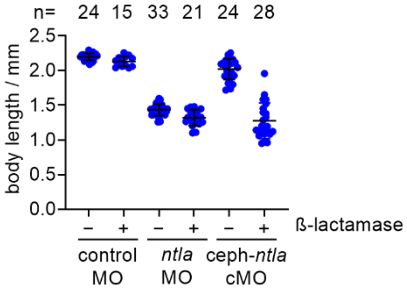

Supporting Figure S8. Body length quantification of cMO-injected embryos. Body length quantification of 24 hpf zebrafish embryos injected with indicated MO (200 pg) alone or co-injected with ß-Lactamase mRNA (400 pg). Body length was measured by using the “straight line” tool in ImageJ and measuring from the top of the embryo’s head to the tip of the embryo’s tail. Bars represent average body lengths ± standard deviation for the respective experimental group.

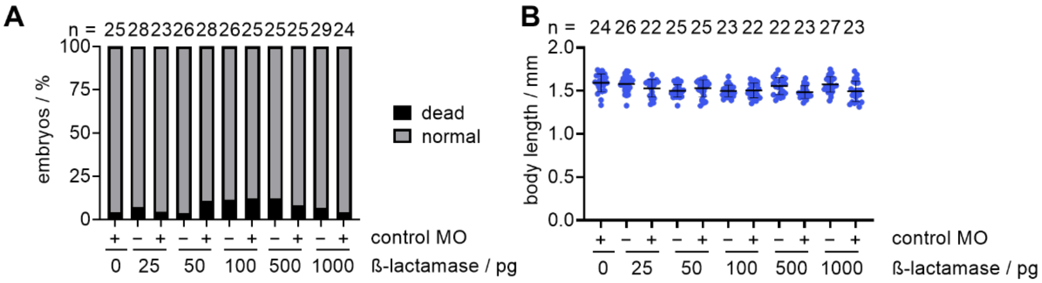

Supporting Figure S9. Toxicity analysis of ß-lactamase mRNA-injected embryos. A) Toxicity analysis and B) body length quantification of zebrafish embryos cultured to 24 hpf following microinjection with increasing amounts of ß-lactamase mRNA with and without negative control MO (200 pg).

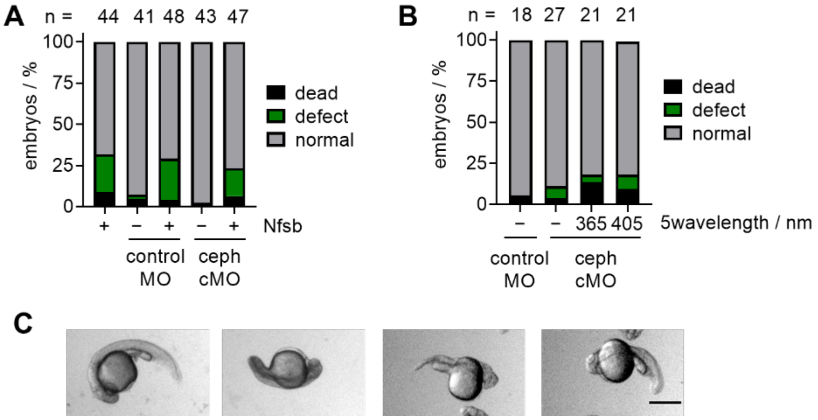

Supporting Figure S10. Orthogonality evaluation of cMO-injected embryos. Phenotypic analysis of 24 hpf zebrafish embryos injected with the indicated MO (200 pg) and A) irradiated with light (365 nm or 405 nm) for 5 minutes or B) co-injected with *Nfsb* mRNA (400 pg). C) Representative embryos demonstrating nonspecific phenotypic defects not consistent with the typical *ntla* phenotype. Scale bar represents 0.5 mm.

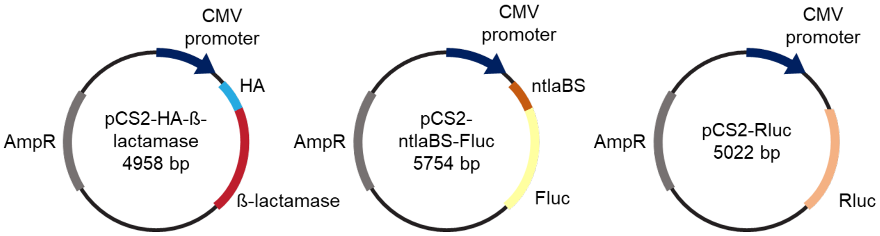

Supporting Figure S11. Plasmid maps. Maps of plasmids prepared for HA-ß-lactamase expression and *in vitro* translation assays.

Supporting Table 1. List of morpholinos and sequences. Terminal modifications are specified. The anti-start codon is indicated in **bold.**

Morpholino	Sequence (5' to 3')
control	CCTCTTACCTCAGTTACAATTTAT
no tail a (ntla)	amine-GACTTGAGGCAGA**CAT**ATTTCCGAT-disulfide amide for sulfhydryl formation

Supporting Table 2. List of gene fragments and primers. Primers were used to generate pCS2-HA-ß-lactamase**.** Restriction sites are indicated in **bold**.

Name	Sequence (5' to 3')
primer P1	ATATC**GGATCC**ATGGGTTCTAGCTATCC
primer P2	TGAAT**CTCGAG**TTACCAGTGCTTGATCAAG
cDNA	ATCGGAAATATGTCTGCCTCAAGTC
mammalian codon optimized HA-ß-lactamase	ATGGGTTCTAGCTATCCGTATGATGTGCCTGACTACGCTA<br>GTTCAGGTCTCGTACCGCGGGGCTCTCATGGTGGGGGTTC<br>TGCTGCCGCCATGCACCCAGAGACACTGGTTAAGGTGAAG<br>GACGCGGAAGATCAGCTTGGTGCCCGAGTGGGCTACATTG<br>AACTTGATCTGAATTCCGGGAAGATCCTTGAAAGTTTCCG<br>GCCGGAAGAAAGATTCCCGATGATGTCCACTTTCAAAGTA<br>CTTCTCTGTGGTGCCGTGCTGTCACGGATAGACGCCGGGC<br>AGGAACAACTGGGCCGAAGAATTCATTATTCCCAGAATGA<br>TCTTGTCGAATACAGTCCGGTTACGGAGAAACATTTGACG<br>GATGGTATGACGGTCCGAGAACTCTGTTCAGCCGCAATCA<br>CTATGAGTGATAACACTGCTGCTAACCTGCTTTTGACCAC<br>CATCGGGGGTCCCAAGGAGCTGACCGCTTTTCTTCATAAC<br>ATGGGCGACCATGTCACACGACTCGACCGATGGGAGCCCG<br>AGCTCAATGAAGCTATCCCTAATGACGAACGAGATACCAC<br>AATGCCTGTGGCTATGGCTACAACCCTCAGAAAGTTGCTG<br>ACCGGTGAGCTTCTCACGCTTGCGTCACGGCAACAGTTGA<br>TTGATTGGATGGAGGCCGACAAGGTAGCGGGTCCTTTGCT<br>GCGATCTGCCTTGCCTGCGGGCTGGTTCATTGCGGATAAA<br>AGCGGGGCTGGTGAGAGGGGATCACGAGGAATCATAGCCG<br>CCCTTGGACCGGACGGAAAACCGAGCAGGATTGTAGTCAT<br>CTACACTACAGGATCTCAAGCTACAATGGACGAGCGCAAC<br>AGGCAGATAGCCGAAATAGGCGCGAGCTTGATCAAGCACT<br>GGTAA

Experimental Protocols

Synthetic Protocols

General chemical methods. All reactions were performed in flame-dried glassware under a nitrogen atmosphere and stirred magnetically. Reactions were followed by thin layer chromatography (TLC) using glass-backed silica gel plates (EMD Millipore TLC Silica Gel 60). Yields refer to pure compounds unless otherwise stated. Starting materials were purchased from commercial sources and used without further purification. Flash column chromatography was performed with silica gel (60 Å, 40-63 µm, 230 x 400 mesh, Sorbtech) as a stationary phase. NMR spectra were recorded on Bruker Ultrashield spectrophotometers.
Compounds **2**,[1] **5**,[2] and **9**[3] were synthesized according to reported procedures.

Synthesis of the cephalosporin linker 8.

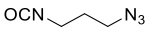

1-Azido-3-isocyanatopropane (3). Compound **2** (50 mg, 0.5 mmol, 0.5 eq) was dissolved in a mixture of DCM (1 mL) and saturated aqueous $NaHCO_3$ solution (1 mL). The mixture was cooled in an ice water bath and diphosgene (240 µL, 2.0 mmol, 2.0 eq) was added. The solution was stirred for 3 h while allowed to gradually warm to room temperature. The DCM layer was separated and washed with saturated aqueous $NaHCO_3$ solution (2 x 1 mL). The DCM layer was dried over sodium sulfate, filtered, and concentrated under reduced pressure. Compound **3** was obtained as a clear oil and was used without further purification (49 mg, 78%). ^{1}H NMR (400 MHz, $CDCl_3$) δ 3.41 – 2.48 (m, 4 H), 1.84 (quin, *J* = 6.42 Hz, 2 H).

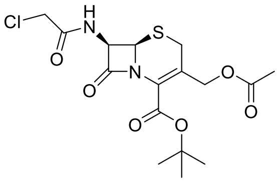

***tert*-Butyl (6*R*,7*R*)-3-(acetoxymethyl)-7-(2-chloroacetamido)-8-oxo-5-thia-1-azabicyclo-[4.2.0]-oct-2-ene-2-carboxylate (6).** Compound **5** (500 mg, 0.91 mmol, 1.0 eq) was dissolved in a mixture of DCM (10 mL) and saturated aqueous $NaHCO_3$ (10 mL). Chloroacetyl chloride (600 µL, 7.61 mmol, 5.0 eq) was added in ten fractions of 0.5 eq each with 15 min intervals between each fraction. The resulting solution was stirred overnight at room temperature. The organic layer was separated, and the aqueous layer was extracted with DCM (3 x 5 mL). The combined DCM layers were washed with brine (10 mL), dried over sodium sulfate (1 g), filtered, and concentrated under reduced pressure. The resulting residue was purified by silica gel flash column chromatography, eluting with ethyl acetate/hexanes (1:4) to yield compound **6** as a white solid (352 mg, 57%). ^{1}H NMR (400 MHz, $CDCl_3$) δ 7.20 (br d, *J* = 9.05 Hz, 1 H), 5.82 (dd, *J* = 9.17, 5.01 Hz, 1 H), 4.99 – 5.09 (m, 2 H), 4.82 (d, *J* = 13.20 Hz, 1 H), 4.12 (s, 2 H), 3.54 – 3.61 (m, 1 H), 3.37 – 3.43 (m, 1 H), 2.09 (s, 3 H), 1.54 (s, 9 H); ^{13}C NMR (101 MHz, $CDCl_3$) δ 170.78, 166.46, 160.34, 127.54, 123.58, 84.18, 63.18, 59.27, 56.93, 42.38, 27.91, 26.51, 20.92; HRMS (ESI) calcd. for $C_{16}H_{20}O_6N_2ClS$ $(M-H)^-$ 403.07251, observed 403.07346.

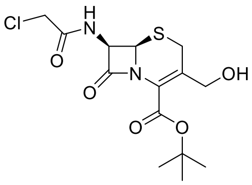

***tert*-Butyl (6*R*,7*R*)-7-(2-chloroacetamido)-3-(hydroxymethyl)-8-oxo-5-thia-1-azabicyclo [4.2.0]-oct-2-ene-2-carboxylate (7).** Compound **6** (300 mg, 0.74 mmol, 1.0 eq) was dissolved in anhydrous THF (2.4 mL) and hexanes (22.6 mL) was added to the THF solution, forming a colorless gel. Lipase acrylic resin (CAL B, Sigma Aldrich Cat. No. L4777) (200 mg, 67 wt%), 2-butanol (1.3 mL, 13 mmol, 18 eq) and 4 Å molecular sieves (300 mg, 1.0 eq) were added to this suspension at room temperature. The resulting mixture was heated to 50 °C and stirred for 5 days.[2] The reaction mixture was filtered, the residue was washed with ethyl acetate (30 mL), and the filtrate was concentrated under reduced pressure. The resulting residue was purified by silica gel flash column chromatography, eluting with ethyl acetate/hexanes (1:1) to yield **7** as a pale-yellow solid (215 mg, 80%). ^{1}H NMR (400 MHz, $CDCl_3$) δ 7.41 (br d, *J* = 8.56 Hz, 1 H), 5.83 (br dd, *J* = 8.74, 4.71 Hz, 1 H), 4.97 (br d, *J* = 4.52 Hz, 1 H), 4.49 (br d, *J* = 12.59 Hz, 1 H), 4.11 (s, 2 H), 3.80 – 4.02 (m, 1 H), 3.51 – 3.68 (m, 2 H), 2.78 (s, 1 H), 1.54 (s, 9 H); ^{13}C NMR (101 MHz, $CDCl_3$) δ 166.63, 163.90, 161.56, 130.18, 126.83, 84.38, 62.15, 59.25, 56.71, 42.37, 27.92, 27.62; HRMS (ESI) calcd. for $C_{14}H_{18}O_5N_2ClS$ $(M-H)^-$ 361.06195, observed 361.05946.

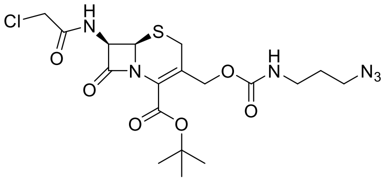

***tert*-Butyl (6*R*,7*R*)-3-((((3-azidopropyl)carbamoyl)oxy)methyl(-7-(2-chloroacetamido)-8-oxo-5-thia-1-azabicyclo[4.2.0]oct-2-ene-2-carboxylate (8).** Compound **7** (50 mg, 0.14 mmol, 1.0 eq) was dissolved in a mixture of DCM (3 mL) and toluene (3 mL). The isocyanate **3** (86 mg, 5.0 eq) was added to this solution at room temperature. Dibutyltin dilaurate (40 µL, 0.069 mmol, 0.5 eq) was added to the reaction mixture, which was stirred overnight at room temperature. The reaction mixture was concentrated under vacuum and the product was purified by silica gel flash column chromatography, eluting with Et_2O/DCM (1:10) to yield **8** as a yellow oil (49 mg, 73%). ^{1}H NMR (400 MHz, $CDCl_3$) δ 7.22 – 7.29 (m, 1 H), 5.81 (dd, *J* = 9.11, 4.95 Hz, 1 H), 4.96 – 5.07 (m, 3 H), 4.82 (br d, *J* = 13.20 Hz, 1 H), 4.11 (s, 2 H), 3.49 – 3.60 (m, 1 H), 3.35 – 3.47 (m, 3 H), 3.22 – 3.35 (m, 2 H), 1.79 (quin, *J* = 6.54 Hz, 3 H), 1.54 (s, 9 H); ^{13}C NMR (101 MHz, $CDCl_3$) δ 166.51, 84.10, 59.25, 56.96, 42.37, 27.94, 26.35; HRMS (ESI) calcd. for $C_{18}H_{24}O_6N_6ClS$ $(M-H)^-$ 487.11611, observed 487.11476.

Synthesis of cephalosporin-*ntla* cyclic cMO.

The cyclic *ntla* cMO bearing the cephalosporin linker was synthesized using a 25-base MO oligomer equipped with 5'-amine and 3'-disulfide modifications purchased from GeneTools, LLC. The sequence can be found in **Supporting Table 1**. Concentrations of all MO and cMO solutions were determined by taking the average of three measurements of the MO solution diluted in 0.1 N HCl (1:10 or 1:20) and measuring absorbance at 260 nm on an ND-1000 NanoDrop spectrophotometer following the recommended protocol from GeneTools.[4]

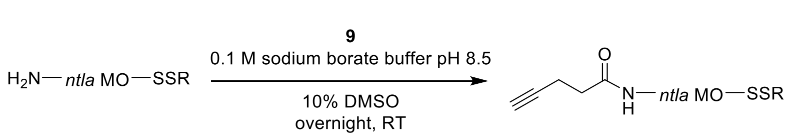

Linear *ntla* MO (100 µL, 1 mM, 100 nmol) was diluted with 0.1 M sodium borate pH 8.5 buffer (894 µL) at room temperature in a 1.7 mL Eppendorf tube and was vortexed briefly to mix. Sodium borate buffer (0.1 M) was prepared by diluting 5 mL of 1 M sodium borate (1 M boric acid, 0.25 M sodium hydroxide) pH 8.5 buffer into 45

mL of water. The NHS-activated alkyne **9** (6 µL, 100 mM in DMSO, 6 eq) was added. The tube was vortexed briefly and the reaction was incubated at room temperature overnight. The next morning, the crude reaction mixture was analyzed by MALDI-TOF mass spectrometry co-spotted with sinapic acid matrix (2:1 matrix/analyte), calculated: 9048.97 Da, observed: 9047. 885 Da (**Supporting Figure S2D**). The alkyne-modified linear *ntla* MO was purified on a Shimadzu LC20AD HPLC (5-60% acetonitrile in 0.1 M TEAA buffer, 20 minute gradient) on an ACE Excel 3 Oligo Beta Test HPLC column (100 x 4.6 mm, identical to EXL-111501046, VWR) using a 1 mL/min flow rate. The MO was monitored via absorbance at 260 nm on a SP-20AD UV/Vis detector and eluted as one peak at ~10 minutes. A sample chromatogram is provided in **Supporting Figure S2D**. The MO fractions containing product were manually collected, pooled, diluted with water, and lyophilized to dryness.

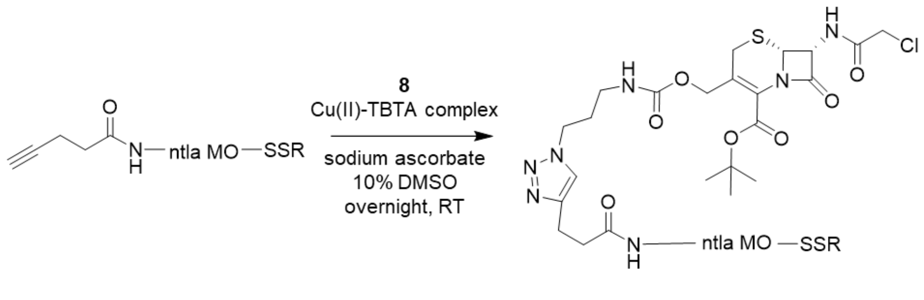

The dry, purified, alkyne-functionalized *ntla* MO was dissolved in water (100 µL) and the concentration was determined on the ND-1000 NanoDrop spectrophotometer following GeneTools recommended protocol (see above, 1:20 in 0.1 N HCl, 265 nm, constant = 34). The typical concentration range can vary depending on the scale of the reaction but is usually between 100-300 µM. The click reaction was performed following a previously reported protocol.[5] The alkyne functionalized *ntla* MO (100 µL, 281 µM, 10.2 nmol) was diluted with water (265 µL) and DMSO (28 µL, 10% final concentration) to a final concentration of 50 µM. The cephalosporin linker **8** (28 µL, 5 mM in DMSO, 51 nmol) was added to the reaction mixture and the reaction was briefly vortexed to mix. In a separate tube, a 5 mM solution of sodium ascorbate was freshly prepared by dissolving solid sodium ascorbate (~ 5-10 mg) in milliQ water (~5.1-10.2 mL, depending on the amount of solid weighed). Then, the aqueous sodium ascorbate solution (112 µL, 5 mM in water, 204 nmol) was added to the reaction mixture which was briefly vortexed to mix. In separate tubes, a 40 mM solution of $CuSO_4$ in water and a 40 mM solution of tris[(1-benzyl-1H-1,2,3-triazol-4-yl)methyl]amine (TBTA) in DMSO were freshly prepared. Then, equal volumes (20 µL) of each solution, the $CuSO_4$ and TBTA solutions, were combined and mixed by pipetting to generate a 20 mM $CuSO_4$-TBTA solution in 50% DMSO. A portion of this 20 mM $CuSO_4$-TBTA solution (28.1 µL, 204 nmol) was added to the linker and alkyne-MO reaction mixture and vortexed briefly to mix. The final concentration of the MO in the reaction mixture was 50 µM. The reaction was incubated overnight at room temperature. The next morning, a small aliquot of the crude reaction mixture was removed and co-spotted with SA matrix and analyzed by MALDI-TOF mass spectrometry as previously described (calculated: 9537.92 Da, observed: 9537.424 Da; **Supporting Figure S2E**). After confirmation of reaction completion, the cephalosporin-*ntla* clicked product was purified by HPLC as previously described. The conjugated MO product elutes as a single, broad peak between 10-13 minutes (**Supporting Figure S2E**). The purified MO fractions were pooled and lyophilized to dryness.

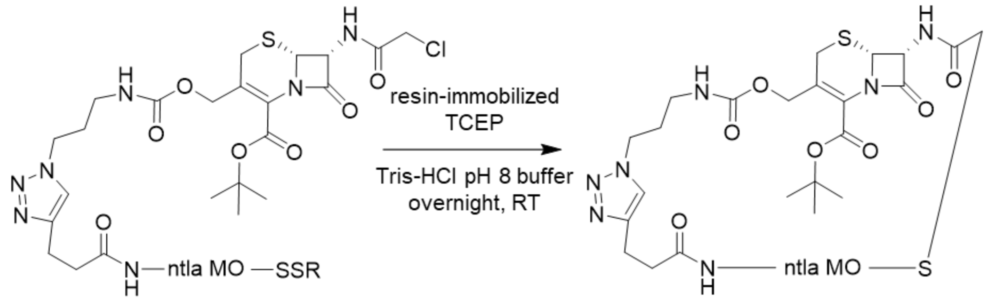

The purified cephalosporin-*ntla* MO conjugate was dissolved in water (150 µL) and diluted with 0.1 M Tris pH 8 buffer (150 µL). Immobilized TCEP slurry (200 µL) was pipetted into a Pierce spin cup filter and mixed with 0.1 M Tris pH 8 (400 µL). The spin cup filter was centrifuged for 2 minutes at 2,000 rpm and the flow-through was discarded. The immobilized TCEP was rinsed twice more with 0.1 M Tris pH 8 buffer (400 µL). Following the final rinse, the resuspended MO solution (300 µL) was added directly to the resin. The tube was inverted to mix and incubated at room temperature overnight. The next morning, the spin cup filter was centrifuged at 2,000 rpm for 2 minutes and a portion of the crude cyclization mixture (2 µL) was co-spotted with sinapic acid matrix (6 µL) and analyzed by MALDI-TOF mass spectrometry (calculated: 9384.29 Da, observed: 9384.991, **Supporting Figure S2F**). Following confirmation of reaction completion, the crude cyclization reaction was purified by HPLC as previously described. The cyclized cephalosporin *ntla* cMO elutes as a broad peak between 9-11 minutes (**Supporting Figure S2F**). Fractions containing purified cMO were pooled and lyophilized to dryness overnight. The cephalosporin *ntla* cMO was subject to additional purifications with NHS-activated agarose and SulfoLink resins to ensure complete removal of any linear MO impurities as described below.

The cephalosporin *ntla* cMO was dissolved in 100 µL of water. To remove any unreacted amine-modified linear MO, the cMO was further purified with NHS-activated agarose (ThermoScientific). Dry NHS-activated agarose (20 mg) was suspended in PBS (400 µL) in a spin cup filter (ThermoScientific). The swollen resin was centrifuged (5000 rpm, 1 minute) and the flow-through was discarded. The cMO solution was diluted 1:1 with PBS (200 µL total volume) and added to the NHS-agarose resin. The tube was inverted slowly to mix and then incubated at room temperature for 30 minutes per the manufacturer's protocol. Following incubation, the tube was centrifuged (5000 rpm, 1 min) and the flow-through was collected. The resin was rinsed with PBS (100 µL), and the flow-through was combined with that from the first spin. The combined elutions were purified on a Shimadzu LC-20AD HPLC using the same conditions mentioned previously. The collected fractions were combined and lyophilized to dryness.

To remove any unreacted, thiol-modified linear MO, the cMO was purified with SulfoLink Coupling Resin (ThermoScientific). Resin slurry (100 µL) was pipetted into a spin cup filter and rinsed with the manufacturer's recommended coupling buffer (50 mM Tris, 5 mM EDTA-Na pH 8.5) twice (400 µL/rinse). Following each rinse, the spin cup filter was centrifuged (1000 rpm, 1 minute) and the flow-through was discarded. The dry cMO was dissolved in water (50 µL) and diluted 1:1 with the coupling buffer (100 µL total volume). The cMO solution was directly added to the resin, and rocked end-over-end at room temperature for 20 minutes, and then allowed to settle on the benchtop for 30 minutes per the manufacturer's protocol. Following incubation, the cMO solution was collected by centrifugation (5000 rpm, 2 minutes). The resin was rinsed once with coupling buffer (100 µL) and centrifuged again (10,000 rpm, 2 minutes) to ensure that all cMO was collected. The flow-through was combined and the cMO was purified on a Shimadzu LC20-AD HPLC using the same conditions as previously mentioned in order to perform a buffer exchange. The collected HPLC fractions were combined and the cMO was lyophilized to dryness.

Following the final purification step, the purified, dried cMO was dissolved in water (40 µL) and the concentration was determined by measuring absorbance at 260 nm on the NanoDrop as previously described. A typical working concentration was ~150-200 ng/µL, and the MO was aliquoted (1-3 µL per aliquot) and stored at −20 °C.

HPLC purification of morpholino conjugates. Morpholino reactions were pipetted into a 200 µL autosampler vial for purification. The sample (typically 50.0 µL at a time) was injected in the HPLC back-to-back until the whole reaction volume was purified. Samples were eluted using a gradient of 5% to 60% acetonitrile in 0.1M TEAA over 20 minutes on a Shimadzu LC-20AD HPLC using ACE3 Excel 3 Oligo Beta Test HPLC column (100

x 4.6 mm, identical to EXL-111501045, VWR) and a 1 mL/min flow rate. Samples were monitored via absorbance at 260 nm on a SP-20AD UV/Vis detector and manually collected as they eluted.

MALDI analysis of morpholino conjugates. The progress of all bioconjugation reactions were monitored by MALDI analysis by removing 1 µL of the reaction mixture and co-spotting it with sinapic acid matrix to dryness on a MSP 96 target ground steel BC MALDI plate. Though a 1:1 sample to matrix ratio was typically used, reactions containing DMSO often required higher ratios (>3:1) and water had to be co-spotted in order to aid in co-evaporation of the DMSO to obtain a dry and crystalline sample for analysis. Analyses were performed on a Bruker Daltonics UltrafleXtreme MALDI-TOF in linear positive mode using 100% power.

Biological Protocols

Cleavage of the cephalosporin linker 8 *in vitro*. Linker **8** was prepared as a 5 mM stock in DMSO and stored at –20 °C as 10 µL aliquots. In two separate reactions, an aliquot of linker **8** was thawed and diluted to 1 mM in 50 mM sodium phosphate buffer pH 8 (40 µL). Recombinant ß-lactamase enzyme (0.5 µL, 1 mg/mL, Novus Biologicals) was added and the reaction mixture was incubated at 30 °C. At the indicated time points (0.5, 1.5, 3, 17, 24 h) a 20 µL aliquot was removed from the reaction mixture and linker cleavage was assessed on a Shimadzu LC-MS-2020 (ESI DUIT, 10 minutes, 100-1000 MHz, 5 µL injection volume) eluting with a gradient of 5-95% acetonitrile + 0.1% formic acid in water.

Cloning of plasmids. Top10 chemically competent *E.coli* cells were used for all cloning. The coding sequence of ß-lactamase was obtained from the sequence specified for the pET15-Beta-Lactamase construct on Addgene (Plasmid # 62729). The coding sequence of an HA tag was inserted upstream of the ß-lactamase sequence (N-terminally tagged) for easy detection by western blot. The entire HA-ß-lactamase coding sequence was optimized for humans using the IDT codon optimization tool. This sequence (**Supporting Table S2**) was then ordered as a gene fragment from Twist Biosciences (San Francisco, CA).[6] The gene fragment was used as a template for PCR amplification with primer P1 and primer P2 (**Supporting Table S2**) in a standard Phusion PCR amplification. PCR amplification was performed in 50 µL reaction volume containing template DNA (50 ng), 5X HF buffer, dNTP mix (200 µM, each), forward and reverse primers (0.5 µM, each), and 1 unit of Phusion polymerase (ThermoScientific). The DNA was amplified using an initial denaturing step (98 °C, 30 s), followed by 34 cycles of denaturing (98 °C, 10 s), annealing (54 °C, 30 s) and elongation steps (72 °C, 45 s). The thermocycler program was completed with a final elongation step (72 °C, 10 minutes), and the samples were stored at 4 °C. The amplicon was resolved on a 0.8% agarose gel stained with ethidium bromide and purified by gel extraction with the E.Z.N.A Gel Extraction kit (Omega BioTek, 101318-972). The pCS2+ vector was digested with BamHI and XhoI (NEB) to generate the backbone fragment. The digest was performed in a 50 µL reaction containing pCS2+-empty (20 µL), 5 µL of 10X CutSmart buffer, and 0.5 µL of each restriction enzyme, XhoI and BamHI-HF. The digest was incubated at 37 °C for atleast 4 hours. The fragments were resolved on a 0.8% agarose gel and purified by gel extraction as previously described. The HA-ß-lactamase insert was then ligated into the digested pCS2+ vector using T4 DNA ligase (NEB). The ligation reaction was performed in a 10 µL reaction containing 100 ng of digested pCS2+ backbone, 65 ng of HA-ß-lactamase insert (3:1 molar ratio), 10X T4 DNA ligase buffer (1 µL), and T4 DNA ligase (0.5 µL). The reaction mixture was incubated overnight in a thermal cycler at 16 °C. The next day, 5 µL of the ligation reaction was transformed into Top10 cells and plated on LB agar plates supplemented with ampicillin (100 µg/mL) and incubated at 37 ºC overnight. Four colonies were inoculated into 5 mL of LB broth containing ampicillin (100 µg/mL) and cultured overnight at 37 °C (250 rpm). Plasmid DNA was purified from bacteria using the GeneJET plasmid miniprep kit (ThermoScientific). Plasmid sequence was confirmed in both direction by Sanger Sequencing (Azenta) using universal sequencing primers (SP6 forward and M13-48REV) provided by Azenta. The pCS2-*ntla*BS-Fluc and pCS2-Rluc constructs were cloned as described previously.[7] Maps of all cloned plasmids are shown in **Supporting Figure S11**.

***In vitro* mRNA synthesis.** The pCS2-HA-ß-lactamase and pCS2-*Nfsb* plasmids were linearized with NotI-HF (NEB).[8] The digest was performed in a 50 µL reaction containing 5 µg of pCS2-HA-ß lactamase or pCS2-*Nfsb*, 5 µL of 10X CutSmart buffer, and 0.5 µL NotI-HF. The digest was incubated at 37 °C for atleast 3 hours. The digested construct was resolved on a 0.8% agarose gel stained with ethidium bromide and purified by gel

extraction following the manufacturer's protocol (E.Z.N.A Gel Extraction Kit, Omega BioTek). Linearized plasmid DNA (1 µg) was transcribed into mRNA using the mMessage mMachine SP6 in vitro transcription kit (Thermo Fisher) following the manufacturer's protocol. The transcription reaction was performed with a total volume of 25 µL, containing 1 µg of digested pCS2-HA-ß lactamase or pCS2-*Nfsb* DNA, 2X NTP/CAP, 10X reaction buffer, and 2 µL of enzyme mix. The reaction mixture was incubated at 37 °C for 5 hours. At the conclusion of the 5 hour incubation, 1 µL of TURBO DNase (provided in the kit) was added to remove the template DNA and the reaction was incubated at 37 °C for an additional 20 minutes. The mRNA was purified through phenol:chloroform:isoamyl alcohol (PCIA) extraction by addition of 30 µL of water and 50 µL of PCIA and vortexing to mix. The water/PCIA mixture eventually settled into two layers and was centrifuged at max speed for 1 minute. The PCIA layer was discarded, and the synthesized mRNA was isolated by ethanol precipitation. The synthesized mRNA was mixed with 5 µL of 3 M sodium acetate and 300 µL of ice-cold 100% ethanol and incubated at −80 °C overnight. The precipitate was pelleted through centrifugation at 4 ºC at max speed for 5 minutes and the ethanol supernatant carefully pipetted off so as to not disturb the pellet and then was discarded. The pellet was washed with 300 µL of ice-cold 70% ethanol and centrifuged at max speed for 5 minutes again. The mRNA pellet was dried on ice for 10 minutes, then dissolved in nuclease-free water to a working concentration of ~ 1 µg/µL. The quality of the transcript was verified on a 0.8% agarose gel stained with ethidium bromide (**Supporting Figure S7**). The mRNA was aliquoted (3 µL per aliquot) and stored at −20 °C.

Cell culture maintenance. All cell culture experiments were performed in a sterile laminar flow biosafety cabinet. HEK293T cells were maintained in Dulbecco's Modified Eagle Medium (DMEM) supplemented with 10% (v/v) fetal bovine serum (FBS) and 1% (v/v) penicillin/streptomycin at 37 °C with 5% CO_2. Cells were used between passage numbers 6 and 25. Cell lines were tested for mycoplasma contamination using the MycoScope Mycoplasma PCR detection kit (Genlantis) every 4 months.

Mammalian cell transfection. HEK293T cells were plated in a 12-well plate pre-treated with poly-D-lysine (MP Biomedicals, 1mg/mL) at a cell density of 150,000 cells/well in DMEM supplemented with 10% fetal bovine serum (FBS) (1 mL/well) and incubated overnight. The next day, cells were transiently transfected with pCS2-HA-ß-lactamase (200 ng/well) using Lipofectamine 2000 (L2k, ThermoFisher) per the manufacturer's protocol. For a 12-well plate, plasmid DNA was diluted in OptiMEM (200 ng plasmid DNA in 100 µL, per well). In a separate tube, L2k was diluted in OptiMEM (4 µL L2k in 100 µL, per well). The DNA and L2k solutions were mixed 1:1 (100 µL each, 200 µL total) and pipetted to mix. The solutions were incubated at room temperature for 20 minutes and then then added to the plated cells (200 µL transfection solution added to 1 mL plated cells) and incubated overnight at 37 ºC. The next morning, the transfection media was removed and replaced with DMEM + 10% FBS (1 mL) and cells were incubated an additional 24 hours. The following day, media was aspirated and cells were carefully rinsed with 500 µL of 1X PBS. RIPA buffer (150 µL) supplemented with 1X HALT Protease Inhibitor cocktail was added to each well and the plate was shaken on ice for 20 minutes. The lysate was removed from each well and pipetted into a 1.7 mL centrifuge tube. Tubes were spun at max speed for 10 minutes at 4 °C. The supernatant was transferred to a new tube and mixed with 4X SDS-loading dye (50 µL, 200 µL total volume in each tube). Samples were boiled at 95 °C for 10 minutes and then stored at −20 °C.

Western blot analysis. Lysates (25 µL) were resolved on a 10% (v/v) SDS-PAGE gel run at 60 V for 20 min and then 150 V for 90 min. Proteins were transferred to a PVDF membrane (80V, 90 min) and the membrane was blocked in blocking buffer (5% [w/v] BSA or non-fat milk in 1X TBS supplemented with 0.1% [v/v] Tween 20 [TBST]) for 2 hours at room temperature while rocking. The choice of blocking buffer was dependent on the manufacturer's specific recommendation for the primary antibody. The membrane was probed with either an anti-HA antibody (Cell Signaling, cat. no. 3742S diluted 1:1000 in 5% [w/v] BSA) or an anti-ß-tubulin antibody (ProteinTech, cat. no. 10068-1-AP, diluted 1:4000 in 5% [w/v] non-fat milk in TBST) at 4 °C overnight while rocking. The following morning, membranes were washed with TBST and the membrane was probed with HRP-linked goat anti-rabbit IgG secondary antibody (ProteinTech, cat. no. SA00001-2, diluted 1:5000 in TBST) for 60 minutes at room temperature while rocking. Following incubation, the secondary antibody was removed and the membrane was rinsed with TBST. Membranes were developed with SuperSignal West Pico PLUS Chemiluminescent Substrate (ThermoScientific, 34580) for 5 minutes, and chemiluminescent signal was imaged on the BioRad ChemiDoc MP Imaging System using the autoexposure settings.

Nitrocefin assay. Nitrocefin (**10**) was obtained as a solid stock from Sigma. Nitrocefin (5 mg) was dissolved in 194 µL sterile-filtered DMSO to generate a 50 mM DMSO stock. The stock was stored as 10 µL aliquots at −80 °C. For activation with recombinant ß-lactamase enzyme (Novus Biologicals), nitrocefin (10 µL from 50 mM stock in DMSO) was diluted in 50 mM sodium phosphate buffer (490 µL) to a final concentration of 1 mM. The nitrocefin solution (50 µL) was pipetted into a transparent, 96-well plate. Where indicated, recombinant ß-lactamase enzyme (0.5 µL, 1 mg/mL) was added to the nitrocefin solution. Absorbance was measured at 390 nm and 486 nm every 2 minutes for 30 minutes on a Tecan M1000 plate reader. The raw absorbance values were plotted as a function of time in PRISM.

For activation in cell lysate, HEK293T cells (40k cells/well) were plated in a white, 96-well plate (pre-treated with poly-D-lysine) in DMEM containing 10% FBS (100 µL) and incubated overnight at 37 °C. The next day, cells were transiently transfected with pCS2-HA-ß-lactamase (100 ng) using Lipofectamine 2000 scaled down from the previously described protocol. The next morning, the transfection media (150 µL) was removed and replaced with fresh medium (DMEM +10% FBS). Cells were incubated for an additional 24 hours. Following incubation, the media was removed, and cells were rinsed with PBS (50 µL). Passive lysis buffer (500 µL, 5X, Promega E1941) was diluted to 1X in milliQ water (2 mL) and 20 µL was added to each well. The cells were lysed at room temperature while shaking for 20 minutes. Following lysis, 50 mM sodium phosphate buffer was added to the lysed cells (30 µL) to a final volume of 50 µL. Nitrocefin (50 µL, 1 mM in 50 mM sodium phosphate buffer) was added to the wells and the reaction mixtures were incubated at room temperature for 2 minutes. Absorbance at 390 nm and 486 nm was measured using a TECAN M1000 plate reader.

Linearization of cMO *in vitro*. Recombinant ß-lactamase enzyme (1 µL, 1 µg/µL) was added to a 5 µL aliquot of cephalosporin *ntla* cMO (25 µM stock, 21 µM final concentration). The aliquot was incubated at 37 °C for 3 hours, after which the enzyme was heat-inactivated for 15 minutes at 65 °C. The aliquot was stored at −20 °C until further use.

Gel-shift assays. Gel-shift assays of cyclic caged MO (cMO) and non-caged morpholinos (MO) were adopted from previous protocols.[9] Samples (10 µL) were diluted with loading buffer (6X Purple Loading buffer, NEB, 2 µL). A Fast Ruler Ultra Low Range ds-DNA Ladder (ThermoFisher Scientific) was used for the gel-shift assays. Reactions (12 µL) were resolved on a 16% Native PAGE in TBE (0.13 M Tris, 45 mM boric acid, and 2.5 mM EDTA, pH = 7.6) running buffer. Following electrophoresis, the gel was stained with SYBR Gold nucleic acid gel stain (Invitrogen, diluted 1:10000 in TBE buffer) for 30 min and then destained in TBE buffer for 15 min. The gel was imaged on a BioRad ChemiDoc MP Imaging system (BioRad) following exposure for 1.037 s. Gel-shift experiments were performed in triplicate.

Standard curve generation

The standard curve was generated by annealing 0.5 µM of *ntla* cDNA with linear *ntla* MO (0.01, 0.025, 0.05, 0.1, and 0.2 µM) in TE/Mg^{2+} buffer (0.01 M Tris-HCl, 10 mM EDTA, 12.5 mM $MgCl_2$, pH = 7.8). A master mix was prepared containing 0.5 µM cDNA in TE/Mg^{2+} buffer (diluting 1:10 from a 5 µM stock). The master mix (18 µL) was aliquoted into PCR tubes, and then 2 µL of the appropriate *ntla* MO solution was added (MO was diluted 1:10 into master mix) and solutions were incubated at 37 °C for 30 min. The resulting solutions were then resolved on a 16% Native PAGE. The gel was imaged on a BioRad ChemiDoc MP Imaging system (BioRad). The resulting bands representing unbound *ntla* cDNA or MO:cDNA heteroduplexes were quantified using ImageJ by drawing rectangles around lanes and analyzed using the plot lanes function. The standard curve was generated using a simple linear regression in GraphPad Prism.

Cephalosporin ntla cMO linearization analysis

For analysis of the linearization of the cephalosporin *ntla* cMO, 125 nM of the cMO was incubated with 0.5 µL of recombinant *β*-lactamase (0.1 mg/mL) for the indicated periods of time (8 µL total reaction). The enzyme was then heat inactivated at 95 °C for 20 minutes. For annealing, 1 µL of cDNA (5 µM) and 1 µL of 10X TE/Mg^{2+} buffer (0.1 M Tris-HCl, 0.01 M EDTA, 125 mM $MgCl_2$, pH = 7.8) were added to the reaction, diluting the cMO to

100 nM. The cDNA and cleaved cMO were incubated at 37 °C for 30 minutes. The resulting solutions were then resolved on a 16% Native PAGE. The gel was imaged on a BioRad ChemiDoc MP Imaging system (BioRad). The resulting bands representing unbound cDNA or the linearized cephalosporin *ntla* MO:cDNA heteroduplex were quantified in ImageJ by drawing rectangles around lanes and analyzed using the plot lanes function. The resulting band density values were extrapolated from the trendline equation from the standard curve (Supporting Figure S3) and used to determine the extent of cephalosporin *ntla* cMO linearization.

Inhibition of *ntla*BS-Fluc translation *in vitro*. *In vitro* translation reactions were prepared using the Rabbit Reticulocyte Lysate Translation System, Nuclease-Treated (Promega, cat. no. L4960) as described previously with slight modifications.[7] A lysate master mix containing lysate, ntlaBS-Fluc mRNA (1 µg), and Rluc mRNA (50 ng/reaction) was prepared and kept on ice. Morpholino solutions were prepared by creating a 5X stock solution according to the desired concentration for the assay. Translation reactions were prepared by mixing 14 µL of the master mix and 3.5 µL of the 5X morpholino solution. The translation reactions were aliquoted into 3 x 5 µL aliquots to generate three independent experiments and incubated at 37 °C for 90 minutes. Following incubation, a 2.5 µL aliquot of each reaction mixture was removed and pipetted into a white, opaque-bottom, 96-well plate. Luminescence was measured on a Tecan M1000 plate reader using the Dual Luciferase Reporter Assay Kit (Promega, E1980). Luciferase assay reagent (40 µL) was added to the well, incubated for 10 seconds, and then firefly luminescence signal was measured. Then, stop-and-glo reagent (40 µL) was added to the same well and *Renilla* luciferase luminescence signal was measured. Luminescence was measured using a 1 second integration time.

For cMO activation experiments, all MO solutions were diluted in water to a concentration of 5 µM. Following incubation with enzyme for the indicated periods of time, the morpholino solutions were added to a translation reaction master mix (14 µL, prepared as specified above) to attain a final MO concentration of 1 µM. The reactions were incubated at 37 °C for 90 minutes and then luminescence was measured as specified above.

Zebrafish aquaculture and husbandry. All zebrafish manipulations were performed in accordance with the Institutional Animal Care and Use Committee (IACUC) at the University of Pittsburgh (protocol no 19075360). Embryos were collected after natural mating of the AB* fish line, maintained under standard conditions, for injection, toxicity studies, and analysis. Embryos were incubated at 29 °C through all experiments. All feeding schedules, temperature control, water quality monitoring, and light cycles were controlled by the veterinary staff at the University of Pittsburgh Division of Laboratory Animal Research (DLAR).

Zebrafish microinjection. Zebrafish microinjections were performed following previously established protocols.[10, 11] Indicated volumes of injection solutions were injected into the yolk of 1-4 cell stage embryos using a World Precision Instruments Pneumatic PicoPump injector. Injection solutions were prepared to a final volume of 5 µL containing phenol red at a final concentration of 0.05% in each solution to be used as a tracer dye for embryo injections. Following injection, embryos were cultured in E3 water supplemented with 0.1% methylene blue at 29 °C. Unfertilized embryos were removed at 3 hpf.

HA-ß-lactamase mRNA microinjections. The HA-ß-lactamase mRNA injection solution was prepared such that the final desired amount of mRNA could be attained following a 2 nL injection. Zebrafish embryos (n = 40) at the 1- to 2-cell stage were injected in the yolk with HA-ß-lactamase mRNA (2 nL) following the microinjection protocol outlined above. Phenotypic analysis of zebrafish embryos was performed as described below.

Zebrafish embryo lysis. Embryos at 24 hpf were pooled, anesthetized in tricaine (16 mg/mL, 6.1 µM, Sigma, cat. no. MS-222), and manually dechorionated using forceps. Embryos were counted and equal numbers per condition were transferred into 1.7 mL Eppendorf tubes. Excess E3 water was removed by pipetting. Embryos were centrifuged at room temperature at 1100 rpm for 10 minutes and residual E3 water was removed. Then, lysis buffer was prepared by diluting 500 µL 5X passive lysis buffer (Promega E1941) in 2.5 mL milliQ water. A volume equal to ~ 2 µL lysis buffer per embryo was added to each tube. Embryos were homogenized on ice using a Pellet Pestle Cordless Motor (Fisher, cat. no. 12-141-361) equipped with an RNase-Free Disposable

Pellet Pestle (Fisher, cat. no. 12-141-364). Following homogenization, samples were analyzed in accordance with the assay under experimentation.

Nitrocefin assay with zebrafish embryo lysate. mRNA injected embryos were removed from the incubator at 24 hpf and transferred to 35 mm petri dishes. The embryos were manually dechorionated using forceps, anesthetized with tricaine (16 mg/mL, 6.1 µM) in E3 water (2.5 mL), and imaged on the Leica MZ16 stereoscope equipped with a QImaging Retiga 1300 camera and captured using the QCapture software. Embryos were pooled and transferred to Eppendorf tubes (n = 41). Excess E3 water was removed by careful pipetting. Embryos were centrifuged at 5,000 rpm at 4 °C for 5 minutes and excess E3 water was removed by pipetting and discarded. Embryos were resuspended in passive lysis buffer (100 µL, Promega E1941) and lysed through homogenization using a Pellet Pestle Cordless Motor (Fisher) equipped with an RNase-Free Disposable Pellet Pestle (Fisher). Embryos were homogenized on ice for 60 seconds and then centrifuged at 5000 rpm for 2 min at 4 °C.

Nitrocefin (10 µL, 50 mM) was diluted to 2 mM in 50 mM sodium phosphate buffer (240 µL) and pipetted into a white, 384-well plate (15 µL). An equal volume of either buffer or embryo lysate (15 µL) was added to each well for a final nitrocefin concentration of 1 mM (30 µL total volume). The absorbance at 389 nm and 486 nm was measured every 2 minutes for 60 minutes on a Tecan M1000 plate reader. The raw absorbance values were plotted as a function of time in PRISM. Initial velocity rates were calculated by performing a linear regression analysis of the absorbance values between 0 and 10 minutes. The slope of the line corresponds to the initial velocity as AU/min. All R^2 values were between 0.96 and 0.99, indicated a good linear fit. The error represents the the standard error of the best fit parameters (i.e., the slope) of the linear regression analysis and was calculated in PRISM.

Western blot analysis of zebrafish embryo lysate. Zebrafish embryos at the 1-to 4-cell stage were injected with 400 pg of HA-ß-lactamase mRNA. Embryos were incubated at 29 ºC to 24 hpf. At 24 hpf, the embryos were pooled into batches of 100, anesthetized in tricaine (16 mg/mL, 6.1 µM), and manually dechorionated using forceps. Embryos were deyolked on ice in 100 µL of embryo deyolking buffer (55 mM NaCl, 1.8 mM KCl, 1.25 mM $NaHCO_3$ in milliQ water) by vigorous pipetting with a P1000 tip until the solution was milky white in color. Deyolked embryos were centrifuged at max speed at 4 °C for 10 minutes, resulting in a relatively big, light brown pellet. Supernatant was discarded. The pellet was washed with deyolking buffer on ice and centrifuged again. The supernatant was removed by pipetting and discarded. The pellet was resuspended in lysis buffer (~2 µL per embryo, 20 mM Tris, 150 mM NaCl, 1 mM EDTA, 1 mM EGTA, 1% Triton X-100) on ice by vigorous pipetting. Once resuspended, the deyolked embryos were lysed by homogenization using the Pellet Pestel Cordless Motor equipped with an RNase-free disposable pellet pestle. Embryos were homogenized on ice for 2 minutes. Once fully homogenized, the lysates were centrifuged at max speed for 20 minutes at 4 °C. The protein concentration of embryo lysates was determined via a Bradford assay.

Samples (50 µg) were resolved on a 12% SDS-PAGE gel (60 V, 20 min, then 180 V, 1 h). Lysates were analyzed via western blot as previously described with an anti-HA antibody (Cell Signaling, cat. no. 2467S) diluted 1:1000 in 5% [w/v] non-fat milk or anti-GAPDH antibody (ProteinTech, cat. no. 60004-1-g) diluted 1:8000 in 5% [w/v] non-fat milk in TBST. The HRP-linked goat anti-mouse IgG secondary antibody (ProteinTech, cat. no. SA00001-1) diluted 1:2500 in TBST for 90 minutes at room temperature. Blots were developed and imaged as described above.

Zebrafish embryo imaging. Embryos at 24 hpf were pooled, anesthetized with tricaine (16 mg/mL, 6.1 µM) in E3 water, and manually dechorionated using forceps. Imaging was performed on a Leica MZ16 stereoscope equipped with a QImaging Retiga 1300 camera and captured using the QCapture software.

ß-lactamase-mediated cMO activation in zebrafish embryos. MO and cMO solutions were diluted to 100 pg/nL in water. MO solutions were heated to 65 °C for 90 seconds to dissociate MO aggregates. Where indicated, injection solutions were diluted with HA-ß-lactamase mRNA (stock ~ 1 µg/µL) to a final concentration of 200 pg/nL. The final volume of all injection solutions was 3 - 5 µL. Embryos at the 1- to 4-cell stage were injected with 2 nL into the yolk directly under the blastomere following the microinjection protocol outlined above.

Unfertilized embryos were removed at 3 hpf. All other zebrafish embryos were incubated at 29 °C until 24 hpf for phenotypic analysis.

Cephalosporin *ntla* cMO orthogonality analysis in zebrafish embryos. MO and cMO solutions were diluted to 100 pg/nL in water as described above. Where indicated, injection solutions were diluted with *Nfsb* mRNA (~1 µg/µL stock) to a final concentration of 200 pg/nL. The final volume of all injection solutions was 3 – 5 µL. Embryos at the 1- to 4-cell stage were injected with 2 nL into the yolk directly under the blastomere following the microinjection protocol outlined above. Where indicated, zebrafish embryos at 1 hpf were irradiated with either a 365 or 405 nm LED (Luxeonstar, Luxeon Z, 675 mW) for 5 minutes by placing the the LED 3 cm above the 35 mm petri dish containing the embryos suspended in E3 water, as previously described.[9] Unfertilized embryos were removed at 3 hpf. All other zebrafish embryos were incubated at 29 °C until 24 hpf for phenotypic analysis.

Phenotypic analysis of zebrafish embryos. All embryos were manually dechorionated at 24 hpf using forceps. Embryos were anesthetized in E3 water supplemented with tricaine (16 mg/mL, 6.1 µM). Bright field images were captured on a Leica MZ16 microscope using the 0.11X zoom (entire group of embryos), 2.0X zoom (representative images of 5 embryos), or 2.5X zoom (single embryo images) with exposure times of 40 ms. For scoring of *ntla* morphant phenotypes, the body length of each embryo was measured from the top of the embryo's head to the tip of the embryo's tail using the straight-line tool in FIJI. Embryo body lengths were scored such that the average length of control MO-injected embryos was set to 1 and the average length of *ntla* MO-injected embryos was set to 0. Parameters for phenotypic scoring were established using the standard deviation of the body lengths for each control group: "normal (wild-type phenotype)" = 0.6 – 1, "weak *ntla*" phenotype = 0.26 – 0.59, "strong *ntla*" phenotype = 0 – 0.25.

^{1}H-NMR Spectra

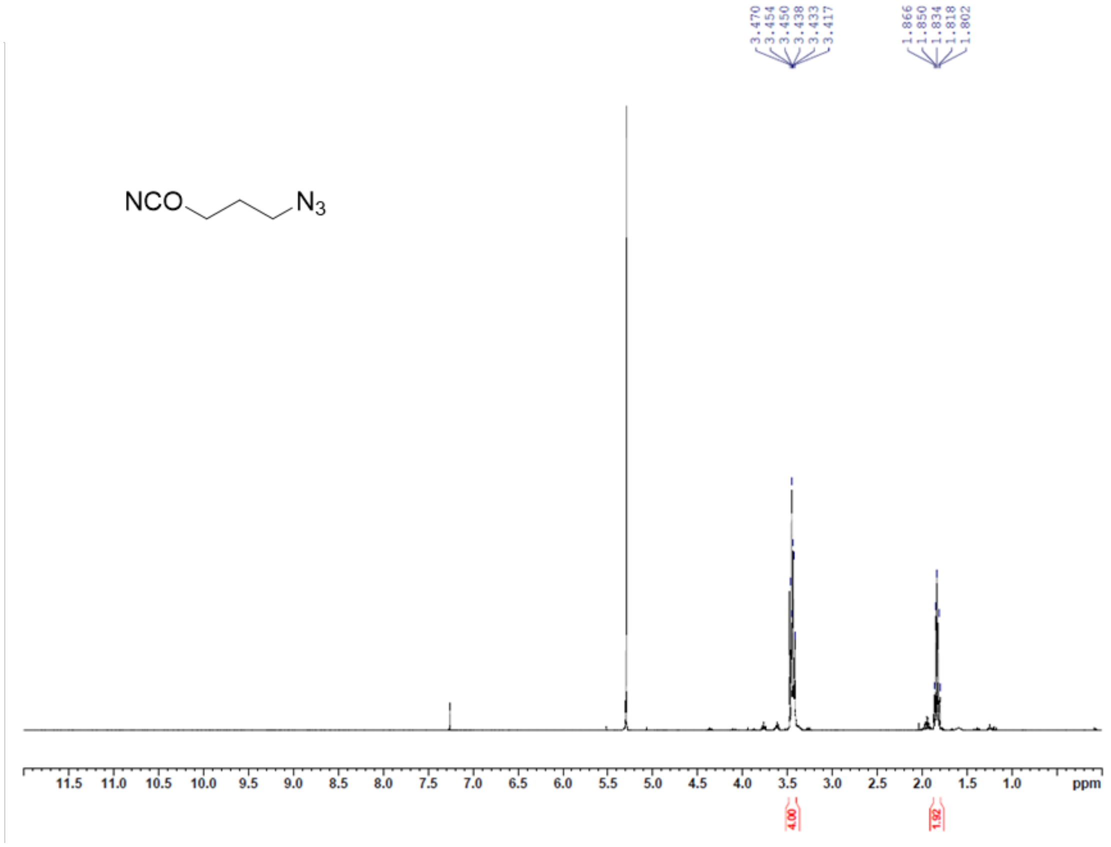

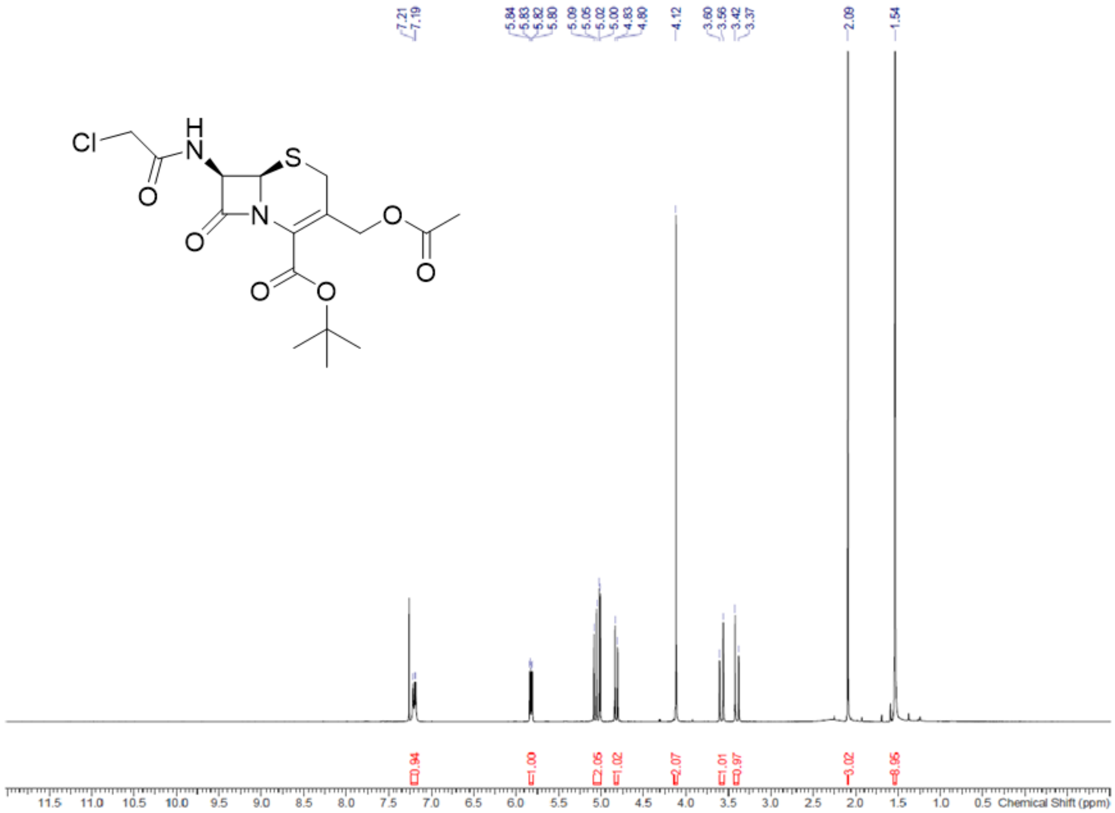

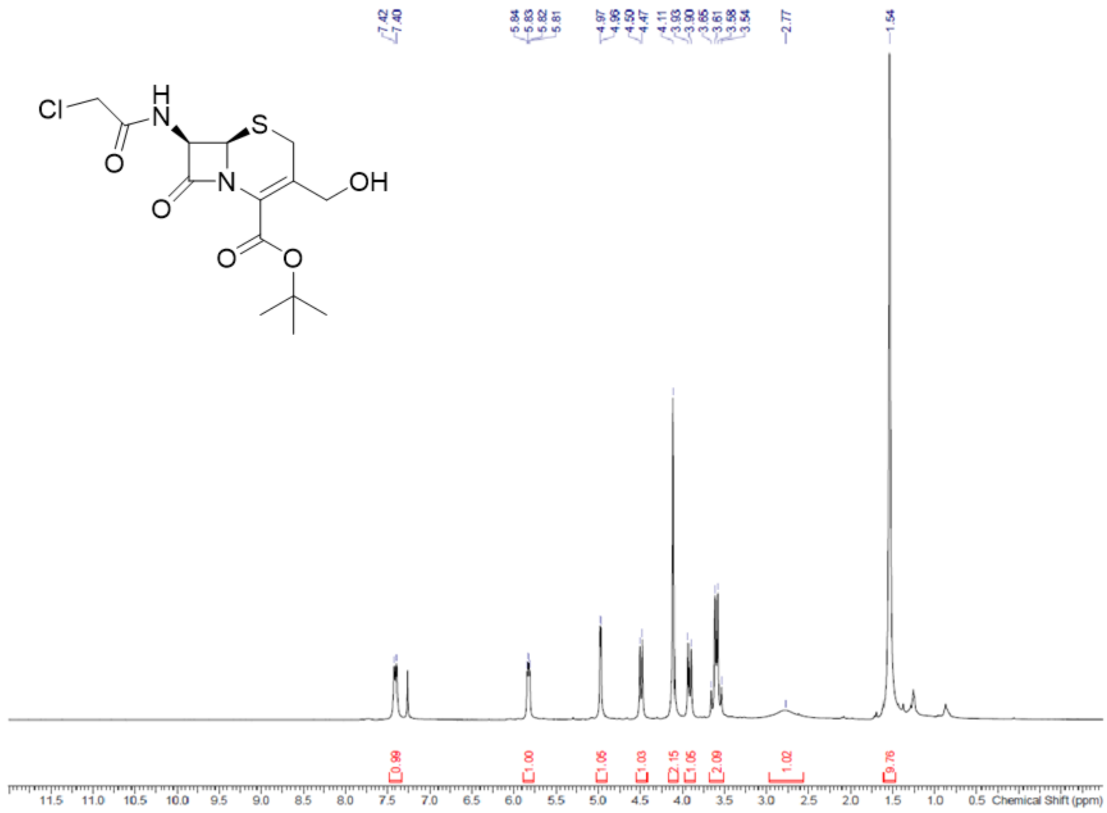
Cl
H
N
S
O
O
N
OH
O
O
Chemical Shift (ppm)

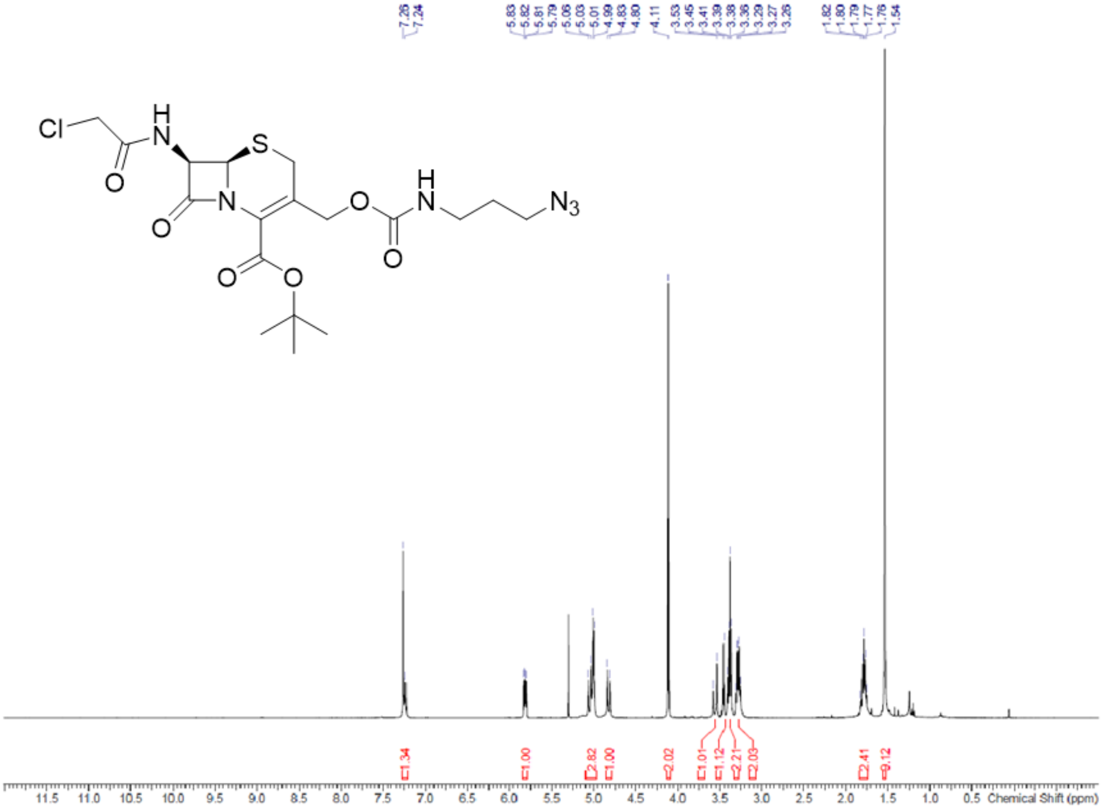
Cl
H
N
S
O
O
N
O
H
N
N_3
O
O
O
Chemical Shift (ppm)

Supporting References

(1) Sarkar, K. M., G.; Chatterjee, K. Dendorn conjugation to graphene ozide using *click* chemistry for efficient gene delivery. *RSC Advances* **2015**, *5*, 50196-50211.

(2) Patterson, L. D.; Miller, M. J. Enzymatic deprotection of the cephalosporin 3'-acetoxy group using Candida antarctica lipase B. *J Org Chem* **2010**, *75* (4), 1289-1292.

(3) Horatscheck, A.; Wagner, S.; Ortwein, J.; Kim, B. G.; Lisurek, M.; Beligny, S.; Schutz, A.; Rademann, J. Benzoylphosphonate-based photoactive phosphopeptide mimetics for modulation of protein tyrosine phosphatases and highly specific labeling of SH2 domains. *Angew Chem Int Ed Engl* **2012**, *51* (37), 9441-9447.

(4) Arbogast, D. *Determining the concentration of your morpholino in solution using the NanoDrop ND-1000 Spectrophotometer.* https://www.gene-tools.com/sites/default/files/NanoDrop%20ND-2.pdf.

(5) Shabanpoor, F.; Gait, M. J. Development of a general methodology for labelling peptide-morpholino oligonucleotide conjugates using alkyne-azide click chemistry. *Chem Commun* **2013**, *49* (87), 10260-10262.

(6) *IDT Codon Optimization Tool.* https://www.idtdna.com/CodonOpt.

(7) Darrah, K.; Wesalo, J.; Lukasak, B.; Tsang, M.; Chen, J. K.; Deiters, A. Small Molecule Control of Morpholino Antisense Oligonucleotide Function through Staudinger Reduction. *J Am Chem Soc* **2021**, *143* (44), 18665-18671.

(8) Schenborn, E. T.; Mierendorf, R. C., Jr. A novel transcription property of SP6 and T7 RNA polymerases: dependence on template structure. *Nucleic Acids Res* **1985**, *13* (17), 6223-6236.

(9) Brown, W.; Bardhan, A.; Darrah, K.; Tsang, M.; Deiters, A. Optical Control of MicroRNA Function in Zebrafish Embryos. *J Am Chem Soc* **2022**, *144* (37), 16819-16826.

(10) Bill, B. R.; Petzold, A. M.; Clark, K. J.; Schimmenti, L. A.; Ekker, S. C. A primer for morpholino use in zebrafish. *Zebrafish* **2009**, *6* (1), 69-77.

(11) Yuan, S.; Sun, Z. Microinjection of mRNA and morpholino antisense oligonucleotides in zebrafish embryos. *J Vis Exp* **2009**, (27).

(12) Rosen, J. N.; Sweeney, M. F.; Mably, J. D. Microinjection of zebrafish embryos to analyze gene function. *J Vis Exp* **2009**, (25).
